# Temperature modulates infection-associated metabolic responses in bloom-forming freshwater diatoms

**DOI:** 10.1093/ismeco/ycag240

**Published:** 2026-08-26

**Authors:** Doris Ilicic, Marine Vallet, Fatemeh Salimi, Anthony T Buaya, Marco Thines, Hans-Peter Grossart

**Affiliations:** Department of Plankton and Microbial Ecology, Leibniz Institute of Freshwater Ecology and Inland Fisheries, Alte Fischerhütte 2, 16775 Neuglobsow, Germany; Department of Biomolecular Chemistry, Leibniz Institute for Natural Product Research and Infection Biology (HKI), Beutenbergstraße 11a, 07745 Jena, Germany; Max Planck Fellow Group Plankton Community Interaction, Max Planck Institute for Chemical Ecology, 07745 Jena, Germany; Senckenberg Biodiversity and Climate Research Centre, Senckenberganlage 25, 60325 Frankfurt (Main), Germany; Department of Biological Sciences, Institute for Ecology, Evolution and Diversity, Goethe University, 60438 Frankfurt (Main), Germany; Senckenberg Biodiversity and Climate Research Centre, Senckenberganlage 25, 60325 Frankfurt (Main), Germany; Senckenberg Biodiversity and Climate Research Centre, Senckenberganlage 25, 60325 Frankfurt (Main), Germany; Department of Biological Sciences, Institute for Ecology, Evolution and Diversity, Goethe University, 60438 Frankfurt (Main), Germany; Department of Plankton and Microbial Ecology, Leibniz Institute of Freshwater Ecology and Inland Fisheries, Alte Fischerhütte 2, 16775 Neuglobsow, Germany; Institute of Biochemistry and Biology, University of Potsdam, 14476 Potsdam, Germany

**Keywords:** primary producers, benthic diatoms, aquatic parasites, metabolomics, global warming, aquatic food webs, oomycetes, chytrids, *Miracula*, *Jonasia*

## Abstract

Warmer waters are reshaping unseen interactions at the base of aquatic food webs, with potential consequences for microalgal bloom dynamics and carbon transfer to higher trophic levels. Two major groups of aquatic parasites—chytrids and oomycetes—infect the same algal host, and changes in temperature can alter the dynamics of these infections and determine their success. Using laboratory-controlled synthetic communities, we investigated the effect of temperature on chytrid and oomycete infection dynamics in the bloom-forming freshwater diatom *Ulnaria ulna*. Our findings reveal complex dynamics, with temperature shaping infection prevalence and host growth differently between the two infection systems. Notably, chytrid infection prevalence increased at lower temperatures, challenging the general applicability of the cold-water refuge theory in this host–parasite system, whereas oomycete infection dynamics showed a contrasting temperature response. Using UHPLC-HRMS analysis, we identified significant metabolic changes associated with parasite infection, while temperature further modulated these responses differently in chytrid- and oomycete-infected cultures. Chytrid infections were associated with metabolites potentially linked to stress- or defense-related responses, whereas oomycete infections exhibited metabolic signatures potentially associated with nutrient scavenging and manipulation of host metabolism. These different metabolic responses suggest that warming not only alters parasite success, but also modifies the biochemical composition of infected host–parasite systems, with possible implications for trophic transfer efficiency and carbon cycling. Together, these findings establish a conceptual framework linking temperature with infection-associated metabolic changes and provide first insights into how temperature affects parasitic infections of microalgae and their biochemical consequences for aquatic food webs.

## Introduction

**Figure 1:**
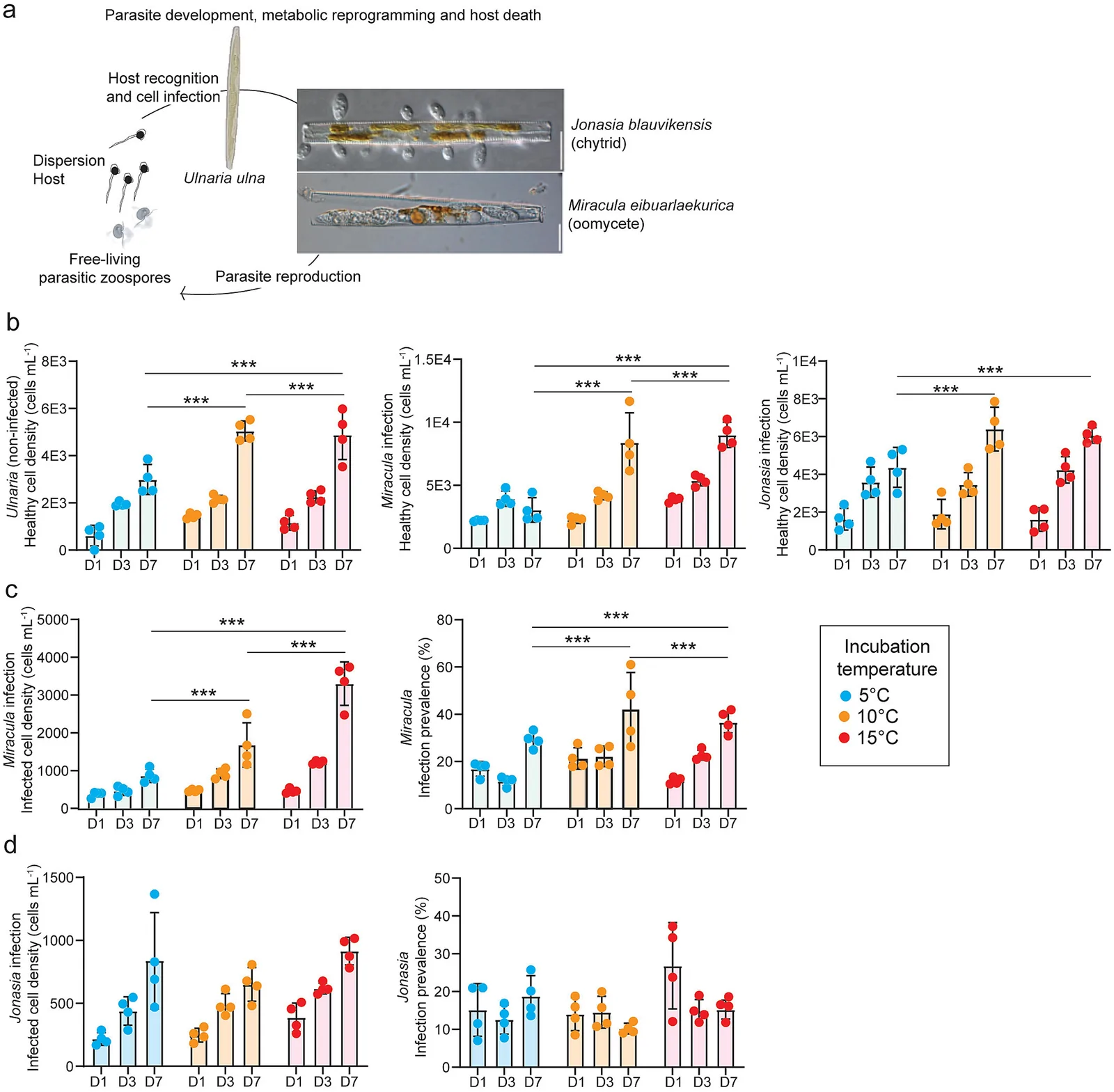
Temperature drives the dynamics of parasitic interactions between *U. ulna* and its associated oomycete and chytrid parasites. The cosmopolitan diatom *U. ulna* is a bloom-forming benthic species that lives as a single cell and can be preyed on by (a) the intracellular oomycete parasite *M. einbuarlaekurica* (*Me*) and the ectoparasitic chytrid parasite *J. blauvikensis* (*Jb*). Cell abundances (cells ml^−1^) of (b) healthy *Ulnaria* cells in non-infected (control) treatment, *Miracula*-infected treatment, and *Jonasia*-infected treatment, (c) infected *Ulnaria* cells in *Miracula* treatment with respective infection prevalence, and of (d) infected *Ulnaria* cells in *Jonasia* treatment with respective infection prevalence at 5°C, 10°C, and 15°C. Values represent mean abundances of four biological replicates, and error bars represent the standard deviation. Statistical analyses of temperature-driven differences were performed using linear mixed-effects models followed by Tukey *post hoc* pairwise comparisons (^***^P < .001). Scale bars in (a) equal 20 μm.

Parasitism has been recognized as a predominant mode of biotic interactions in aquatic ecosystems worldwide [1]. This prevalence highlights its significance in shaping aquatic food webs by affecting population dynamics, biodiversity, and trophic interactions [2]. Although highly abundant in aquatic ecosystems, fungal and fungus-like parasites are often neglected, and their ecology has been poorly studied [3, 4]. However, driven mainly by molecular studies highlighting their ecological importance, scientific interest has significantly increased over the past few years. For instance, chytrids represent a group of zoosporic fungi belonging to the phylum Chytridiomycota that are ubiquitously found in aquatic systems. As parasites of many microalgal species, chytrids strongly influence bloom dynamics [5, 6], nutrient transfer [7, 8], and aquatic food-web structure. However, there is a second similarly large group of organisms, the Oomycota, which plays an equally important role in marine and limnic ecosystems, but has been largely neglected until recently [9]. Oomycetes are fungus-like stramenopiles that also parasitize aquatic microalgae and remain comparatively understudied in aquatic ecosystems [4, 10, 11]. Unlike many chytrids, they typically develop a holocarpic thallus within a host cell and recruit the entire host cytoplasm for their reproduction [12]. In both groups, infections are initiated by motile zoospores that locate algal hosts, encyst on the host surface, and develop into reproductive structures that exploit host resources and ultimately release new zoospores, completing the infection cycle and killing the host (Fig. 1a; they thus function as parasitoids).

Molecular mechanisms underlying algal infections by fungi and oomycetes are still poorly understood, partly because of challenges in establishing a reliable co-culture pathosystem suitable for detailed physiological studies in the lab, and partly because the complex chemistry of aquatic ecosystems hinders the detection of signals from bioactive metabolites at low concentrations. Experimental studies on diatoms have shown increased production of polyunsaturated aldehydes (PUAs) under stress conditions in the case of *Thalassiosira rotula* [13, 14], whereas oxylipins were reported in the diatom *Cerataulina pelagica* [15]. These compounds may play an essential role in algal defense mechanisms [16]. Studies investigating the molecular mechanisms and underlying compounds that drive chytrid and oomycete infections are just starting to emerge. One of the few such studies has shown that the oomycete parasite *Lagenisma coscinodisci,* infecting the diatom *Coscinodiscus granii,* induces the production of algal alkaloids, specifically β-carboline and 2,3,4,9-tetrahydro-1H-β-carboline-3-carboxylic acid during the infection, thereby inhibiting host cell division and increasing the overall infection rate [17]. In addition, other studies using the same pathosystem have identified the tetrapyrrole derivative pheophorbide *a* [18] as one of the metabolic predictors of infection-associated chlorophyll degradation, and the glycerophospholipid lysophosphatidylcholine [19], which is potentially involved in host defense and infection-related cellular signaling.

In contrast, the molecular and metabolic changes that occur during algal infections by chytrids in aquatic ecosystems remain to be unraveled. Although several studies have confirmed that photosynthetic exudates serve as chemical signals driving the chemotaxis of chytrid zoospores [20–22], the molecular mechanisms underlying these infections remain poorly understood. One study using phytochemical screening in four chytrid-diatom tandem cultures (*Chytridium* sp.-*Navicula* sp., *Rhizophydium* sp.-*Nitzschia* sp., *Rhizophydium* sp.-*Rhizosolenia* sp., *Rhizophydium* sp.-*Chaetoceros* sp.) reported putative antimicrobial compounds (e.g., flavonoids) that contributed to diatom defense against chytrid zoospores [21]. Moreover, host susceptibility to parasite infections is frequently dependent on abiotic environmental conditions, which influence the population dynamics of both hosts and parasites across various systems and shape the intensity and nature of host–parasite interactions [23–25].

In the past decade, global change has significantly contributed to temperature increases in aquatic ecosystems worldwide [26], with particularly pronounced effects observed in coastal areas of Arctic and sub-Arctic regions [27]. The balance of marine food webs in these areas relies heavily on the primary production of benthic diatoms [28, 29]. Therefore, understanding the response of parasites associated with these organisms to warming is crucial for predicting the broader ecological impacts of global climate change on these ecosystems.

Here, we used two recently established stable laboratory Arctic pathosystems consisting of chytrid- and oomycete-infected benthic diatom *Ulnaria ulna*, namely *Jonasia blauvikensis* (*Jb*) and *Miracula einbuarlaekurica* (*Me*) [30], to: (i) describe how temperature variation affects chytrid and oomycete infection processes, (ii) identify infection-associated metabolites that differ between chytrid- and oomycete-infected cultures, and (iii) determine how temperature variation influences the metabolomic profiles of the host–parasite systems.

## Materials and methods

### Experimental setup

Two pathogen model systems described by Salimi et al. [30] were used, comprising the diatom host *U. ulna* agg. and its common parasites: the chytrid *J. blauvikensis* (strain Chylce1g) and the oomycete *M. einbuarlaekurica* (strain D3p11), both isolated from freshwater streams in Iceland. Salimi et al. [30] reported that both parasites frequently co-occur in the same freshwater habitats and are present year-round across multiple Icelandic streams. *U. ulna* is a widespread pennate freshwater diatom [31, 32] that predominantly occurs in benthic biofilms but may also occur in the pelagic plankton [33]. Species of the genus *Ulnaria* can form dense blooms and often serve as hosts for chytrid parasites in aquatic ecosystems [34]*.*

The experimental setup consisted of three treatments per tested temperature. Treatment 1 (Uln) consisted of non-infected *Ulnaria* cells and acted as a control for *Ulnaria* population growth; treatment 2 (*Jb*) consisted of chytrid-infected, and treatment 3 (*Me*) of oomycete-infected *Ulnaria* culture. Experiments were performed in batch cultures, and each treatment and infection experiment was replicated four times. Pregrown *Ulnaria* cultures were split into 36 × 20 ml aliquots into 500 ml cell culture flasks containing 100 ml f/2 medium. This ensured comparable initial cell densities across replicates and allowed several generations of population growth before nutrients became limiting. Of these, 12 were inoculated with 5 ml of a 1-week-old chytrid-infected culture (with ~52% infection prevalence) and 12 with a 1-week-old oomycete-infected culture (with ~58% infection prevalence) to initiate the infections. The infection prevalence was 18% in both infected treatments on Day 0. Effects of temperature were tested by incubating the treatments in incubation chambers at 5, 10, and 15°C. For each temperature treatment, both infected and non-infected replicate cultures were maintained. The temperature of 10°C represents the optimal cultivation condition for host–parasite co-cultures under laboratory conditions and was therefore used as the control temperature, while the tested temperatures (5 and 15°C) were defined as the treatments to simulate environmental scenarios of low and elevated temperature conditions for Arctic and sub-Arctic freshwater systems. The experiment was conducted over 7 days, as this was previously determined to be the duration of two infection cycles for both parasites. The positions of the replicates were randomly changed every other day to eliminate any location effect. Light conditions were kept constant with 24 h light, mimicking the Arctic summer. Each replicate was sampled after 1, 3, and 7 days for non-infected and infected *Ulnaria* population cell counts and calculation of parasite infection prevalence. Samples for metabolomic extraction were collected on Day 7.

### Cell counts, infection prevalence, and statistical analysis

Diatom cell abundance and infection prevalence were analyzed from 2 ml subsamples, which were preserved with Lugol and stored at 4°C in the dark. Before counting, 25 μl of 3% Na_2_S_2_O_3_ was added to the sample to remove the color of the Lugol’s solution. Cells were counted at 400× magnification under an inverted microscope (Nikon Eclipse Ti2) in Utermöhl plankton chambers (Hydrobios, Germany). At least 300 cells were counted in each sample. Normality was tested using the Shapiro–Wilk test, and data variance was tested using Bartlett’s test. Parasite infection prevalence was calculated within each group by temperature and parasite type as the number of infected cells divided by the total number of *Ulnaria* cells. To evaluate the effects of temperature, parasite treatment, and sampling time on host cell abundance, infected cell abundance, and infection prevalence, linear mixed-effects models were fitted using the *lme4* and *lmerTest* packages in R (v. 4.6.0) [35, 36]. Temperature, parasite treatment, and timepoint were included as fixed factors, while replicate identity was included as a random factor to account for repeated sampling over time. Type III ANOVA with Satterthwaite approximation was used to assess the significance of fixed effects and interaction terms. *Post hoc* pairwise comparisons of estimated marginal means were performed using the *emmeans* package with Tukey-adjusted *P*-values [37]. Statistical significance was assessed at α = 0.05.

### Metabolic extraction

After 7 days of incubation, 100 ml of culture was filtered under vacuum on 47 mm GF/C microfiber filters (Whatman) using a filtration unit. Prior to filtration, culture flasks were vigorously shaken to detach cells from the flask surface. Following filtration, the flask surfaces were visually inspected and only negligible numbers of attached cells remained. The GF/C filters were directly transferred to 2 ml safe-lock Eppendorf tubes and stored at −80°C until further processing. Then, 1.2 ml of cold methanol (99.8%, anhydrous, Sigma-Aldrich, Germany) was added to the tubes, and the cells were disrupted by sonication in an ultrasound bath for 15 min. Organic phases were transferred into new glass vials and centrifuged for 20 min at 14 000 rpm at 15°C. The resulting supernatant was transferred into 1.5 ml glass vials and dried using a Vacufuge® plus vacuum concentrator (Eppendorf). The extracts were taken up with 100 μL methanol, homogenized with a vortex, transferred to a 1.5 ml tube with a glass Pasteur pipette, and centrifuged for 20 min at 14 000 rpm. Subsequently, 30 μL of each sample was transferred into 1.5 ml Liquid Chromatography-Mass Spectrometry (LCMS) glass vials with inserts. To prepare the QC (quality control) sample, 10 μL of each sample was combined into the QC pool. Blank samples consisted of f/2 culturing medium and were prepared as the extract samples, but were not added to the QC sample.

### Chromatography and mass spectrometry analysis

Samples for UHPLC-HRMS analysis were measured with a Dionex UltiMate 3000® (Dionex, Germany) coupled with a Q-Exactive Plus Orbitrap mass spectrometer (Thermo Scientific, Germany). Two distinct chromatographic protocols were implemented to identify a wide range of metabolites, including C18 and ZIC-HILIC columns. Accucore C-18 column (100 × 2.1 mm, 2.6 μm, Thermo Fisher Scientific, Germany) was used for the chromatographic separation of non-polar metabolites. The sample injection volume was 1 μL. Mass spectrometry was conducted in both positive and negative polarities with a scan range of *m/z* 100 to 1500 at a peak resolution of 70 000. The MS/MS spectra of precursor ions selected with an inclusion list were obtained from the pooled QC sample. Detailed information on chromatographic conditions and mass spectrometry settings is provided in the Supplementary Materials. Datasets are publicly available in the MassIVE spectral database under MassIVE Accession numberTeX MSV000097103.

### Data transformation, statistical analysis, and metabolites identification

Statistical analysis was conducted on the different data matrices using MetaboAnalyst 6.0 [38]. Significantly upregulated metabolites identified using statistical analysis were further investigated by acquiring MS/MS spectra using ddMS experiments on QC pool samples (Supplementary Figs S3–S22). To identify and confirm significant features, the MS/MS spectra of selected ions were compared using SIRIUS (v5.8.3) and CSI:FingerID. Based on the tree fragmentation score and the percentage returned by the CSI:FingerID tool, putative identifications of unknowns were performed. MS/MS raw spectra are available in the Files tab. Assignment of identity with a confidence level was conducted using a 70% similarity threshold in SIRIUS and a minimum cosine score of 0.7 in Global Natural Products Social Molecular Networking (GNPS). The resulting network is publicly available in the MassIVE database under MassIVE Accession number MSV000097102. Mirror matches with public database MS/MS spectra are shown in Supplementary Figs S3, S5, S6, S8, S10, and  S16. Abundance plots using peak intensities normalized to cell count were generated in GraphPad Prism version 10.0.0 for Windows (GraphPad Software, Boston, Massachusetts, USA).

## Results

### Infection prevalence and diatom growth response to temperature changes

For cell enumeration, we distinguished between non-infected and infected *U. ulna* (syn. *Synedra ulna*) cells. Non-infected *Ulnaria* cells were recognized as healthy when cells were intact, displaying chlorophyll autofluorescence without sporangia. Infected *Ulnaria* cells included those with intracellular oomycete thalli of *M. einbuarlaekurica* (Fig. 1a) or attached chytrid sporangia of *J. blauvikensis* (Fig. 1a), as well as post-infected and decaying cells without chlorophyll autofluorescence and with remains of chitinous or glucanous cell walls indicating previous parasite infections. Chlorophyll autofluorescence was used qualitatively to identify intact photosynthetic cells and to discriminate them from infected, senescent, or damaged cells in mixed algal populations [39]. Linear mixed-effects models revealed significant interaction effects between temperature, parasite treatment, and sampling time on infected cell abundance and infection prevalence (Type III ANOVA, *P* < .001; Table S2), indicating that infection dynamics differed between chytrid and oomycete treatments across temperatures and over time. In the non-infected control treatment (Uln), the total abundance of non-infected *Ulnaria* cells increased with increasing temperature, indicating healthy growth in the absence of a parasite (Fig. 1b). *Ulnaria* growth was significantly lower at 5°C compared to 10°C and 15°C, whereas no significant difference was observed between 10°C and 15°C (Tukey *post hoc* test, *P* < .01; Table S3).

In the oomycete-infected (*Me*) treatment, the number of non-infected *Ulnaria* cells at 5°C increased until Day 3 and decreased thereafter. At 10°C and 15°C, the numbers increased continuously throughout the experiment (Fig. 1b). This pattern may reflect a longer life cycle of the parasite, which could allow an initial increase in host cells before being counterbalanced as the parasite produces more offspring per cycle. However, it also highlights observed temperature-specific differences in host–parasite dynamics. The number of infected *Ulnaria* cells in the *Me* treatment increased over 7 days, with significant differences observed between all tested temperatures (Fig. 1c). *Post hoc* Tukey comparisons showed significantly lower infected-cell abundance at 5°C compared to 10°C and 15°C. Oomycete infection prevalence at Day 7 was significantly higher at 15°C than at 5°C and 10°C (*P* < .001; Fig. 1c).

In the chytrid-infected (*Jb*) treatment, the abundance of infected *Ulnaria* cells did not differ significantly across temperatures (Fig. 1d), indicating that low temperature did not reduce chytrid infectivity. However, *post hoc* Tukey comparisons revealed significantly lower increases in non-infected *Ulnaria* cells at 5°C compared to 10°C and 15°C (*P* < .001 for both comparisons; Fig. 1b). Because the abundance of non-infected host cells changed differently across temperature treatments, the prevalence of chytrid infection increased from 15% to 19% at 5°C, whereas it decreased from 14% to 11% at 10°C (Fig. 1d). At 15°C, however, chytrid infection prevalence initially increased from 18% to 27%, then declined to 15% by Day 7. Detailed cell count data and statistical outputs are provided in Supplementary Tables S1–S3.

### Metabolic alterations associated with temperature changes and specific metabolic features associated with parasitic infections

**Figure 2:**
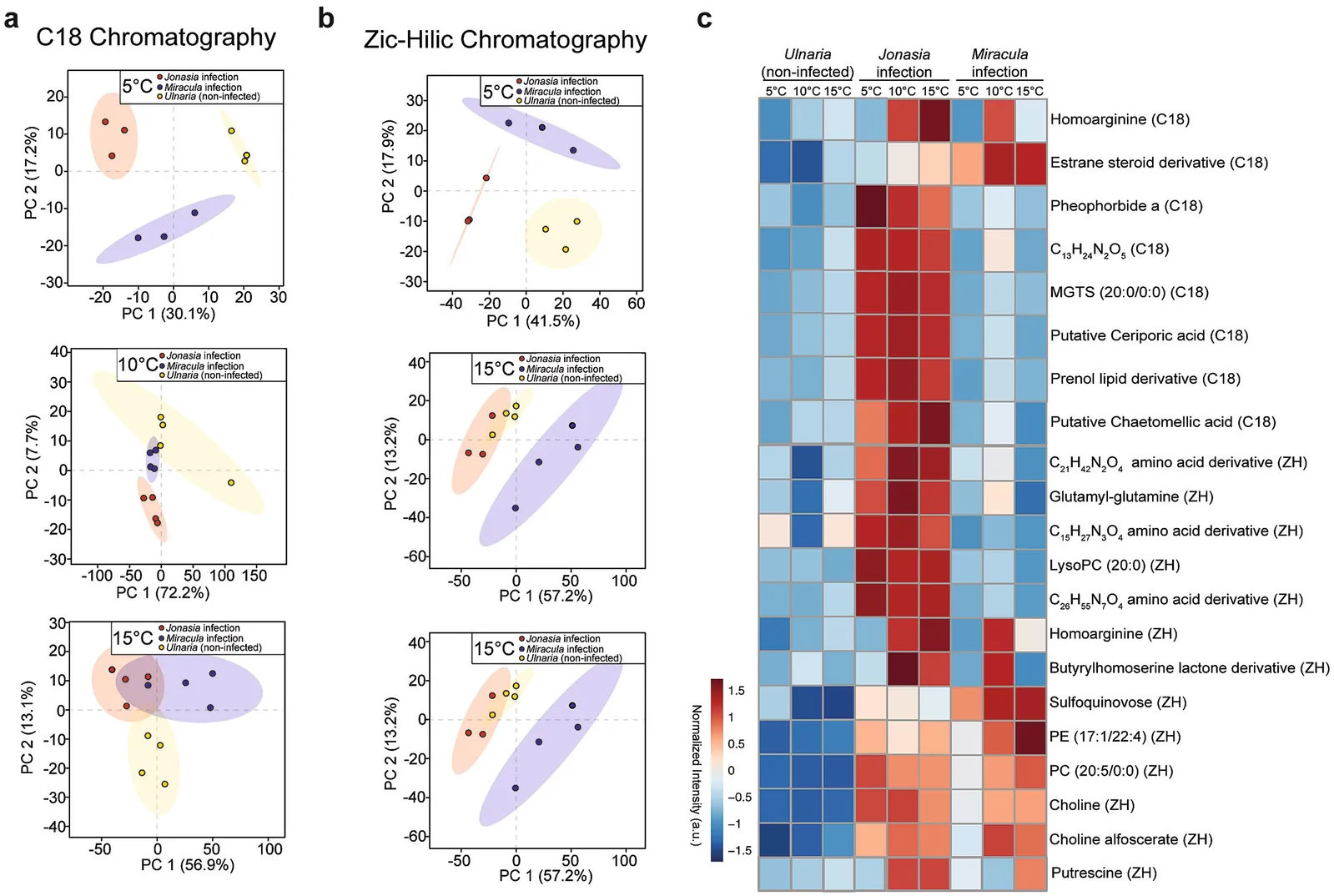
Metabolomic alterations of the diatom *U. ulna* infected by oomycete and chytrid parasites under different temperature conditions. Principal component analysis on cellular extracts of non-infected (*Ulnaria*), chytrid-infected (*Jonasia*), and oomycete-infected (*Miracula*) *U. ulna* cells was conducted on UHPLC-HRMS datasets acquired using two distinct columns, i.e., (a) C18 and (b) ZIC-HILIC (ZH), to target polar to non-polar compounds. Distinct metabolomic profiles were highlighted between the cell extracts, regardless of the incubation temperature (5°C, 10°C, and 15°C). (c) Identification of significant metabolites associated with chytrid and oomycete infection in *U. ulna* cell extracts. The identification is based on spectral similarity matching of MS2 fragmentation with MS2 spectra from public databases. Peak intensities were normalized to cell counts, log-transformed, and Pareto-scaled.

Preliminary experiments showed that high infection prevalence for both parasites was detected on the seventh day of incubation, while many healthy host cells were still present. Therefore, this time point was chosen to study the cellular metabolic response of the host *U. ulna* subjected to chytrid (*Jb*) or oomycete (*Me*) infections. Because metabolomic sampling was performed only at Day 7, the detected profiles should be interpreted as integrated endpoint signatures of infection progression rather than temporally resolved host responses. An untargeted metabolomics approach was employed using ultra-high-performance liquid chromatography-high-resolution mass spectrometry (UHPLC-HRMS). Non-infected and infected host cell extracts were analyzed using C18 and ZIC-HILIC columns, and the polarity switch was used to cover a broad range of metabolites. Additionally, we used an RP18 column to target lipids, oxylipins, and fatty acids, analyzing the samples incubated at 10°C and 15°C. Peak intensities were normalized to total cell abundance (Supplementary Table S4), yielding a matrix with 6600 signals (putative elemental composition, *m/z*, retention time) for analysis with a C18 column and 5425 signals for analysis with a ZIC-HILIC column. A total of 6609 features were detected during the UHPLC-HRMS analysis using the RP18 column. Metabolic profiles of extracts from non-, chytrid-, and oomycete-infected *Ulnaria* cells were clearly distinguished by principal component analysis (PCA) with total principal component variance ranging from 47.3% to 79.9% (Fig. 2a and b). Both temperature and parasite infection altered the cellular metabolic profiles of the diatom *U. ulna*, with the detection of significant features associated with one or both parasite infections. The most significant discrimination between non-infected and infected cellular extracts was observed at the lowest temperature (e.g. 5°C) for both C18 and ZIC-HILIC analyses (Fig. 2a and b). While cell-abundance normalization minimizes differences due to biomass, the contribution of temperature-induced metabolic shifts cannot be fully excluded.

**Figure 3:**
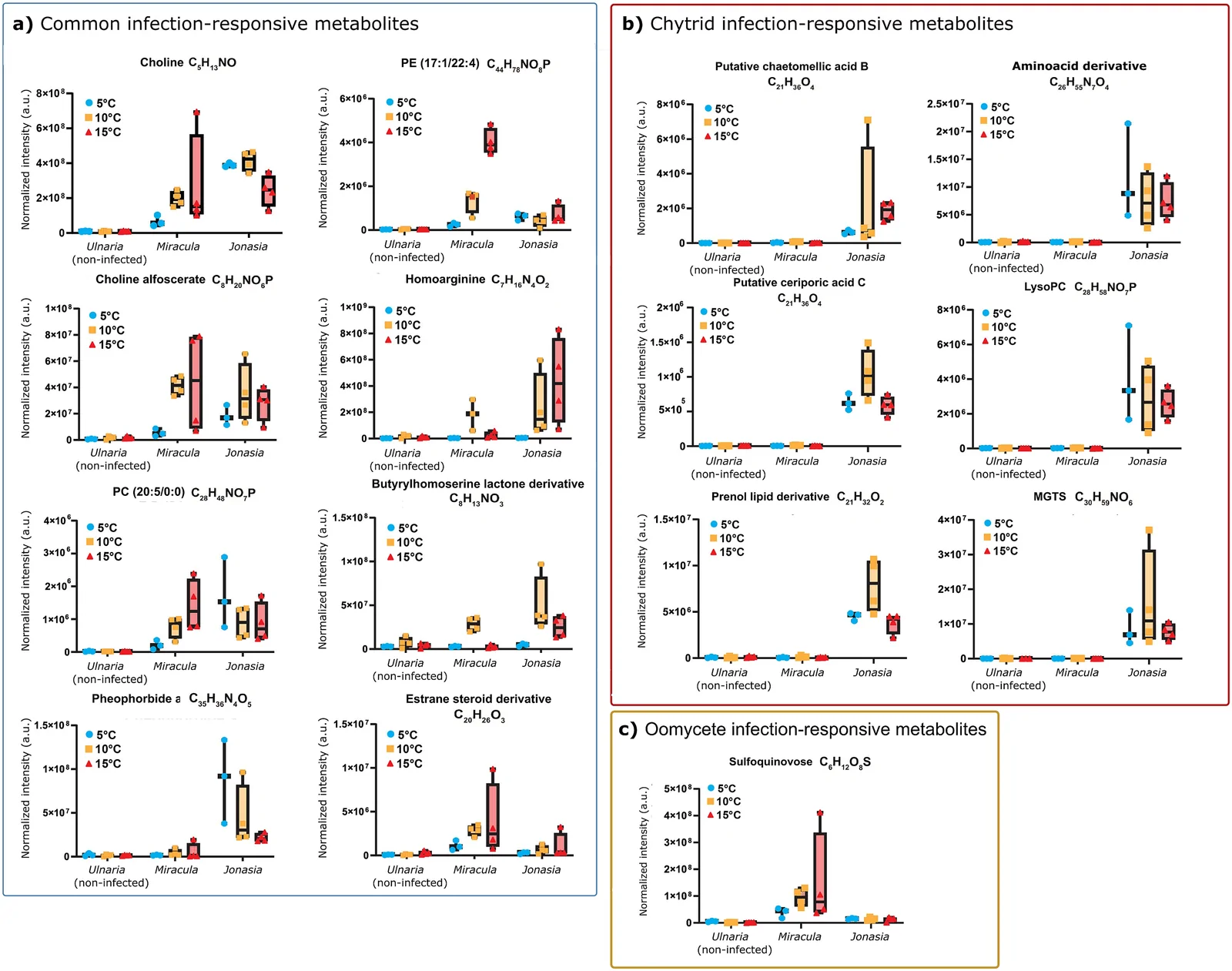
Temperature changes and parasite species are associated with alterations in algal metabolites. Metabolites related to both chytrid and oomycete infection (a), specific for chytrid infection (b), or oomycete infection (c) during temperature changes. The identification is based on spectral similarity matching of MS2 fragmentation with MS2 spectra from the GNPS public database, and fragmentation analysis was done with SIRIUS CSI:FingerID. Intensities of peak areas were normalized based on cell counts, log-transformed, and Pareto-scaled.

Among the signals that were elevated in infected extracts, 27 metabolites were tentatively elucidated with high-resolution MS/MS, of which 20 were further confirmed at level 2 of MS confidence (Fig. 2c). For samples incubated at 10°C and 15°C, the RP18 column enabled the detection of prostaglandins, eicosanoid derivatives, and long-chain fatty acids (Supplementary Figs S1 and S2). Some of the identified metabolites are likely of parasite origin, as they were not detected in non-infected *Ulnaria* cell extracts or might be produced *de novo* during host cell infection, considering that metabolomic analyses were performed on infected host cultures rather than physically separated host and parasite biomass (Fig. 2c). Several amino acid derivatives, lipids, choline, and glycerophosphorylcholine (choline alfoscerate), PE (17:1/22:4), and PC (20:5/0:0) were among these infection-related metabolites (Fig. 3a). These metabolites were associated with parasite-infected treatments and may reflect infection-associated metabolic responses, and no significant differences in their relative abundance were observed between the applied incubation temperatures. Moreover, the detection of certain lipids was specifically associated with chytrid infection, such as MGTS, putative ceriporic acid and chaetomellic acid, and a prenol lipid derivative (Fig. 3b).

**Figure 4:**
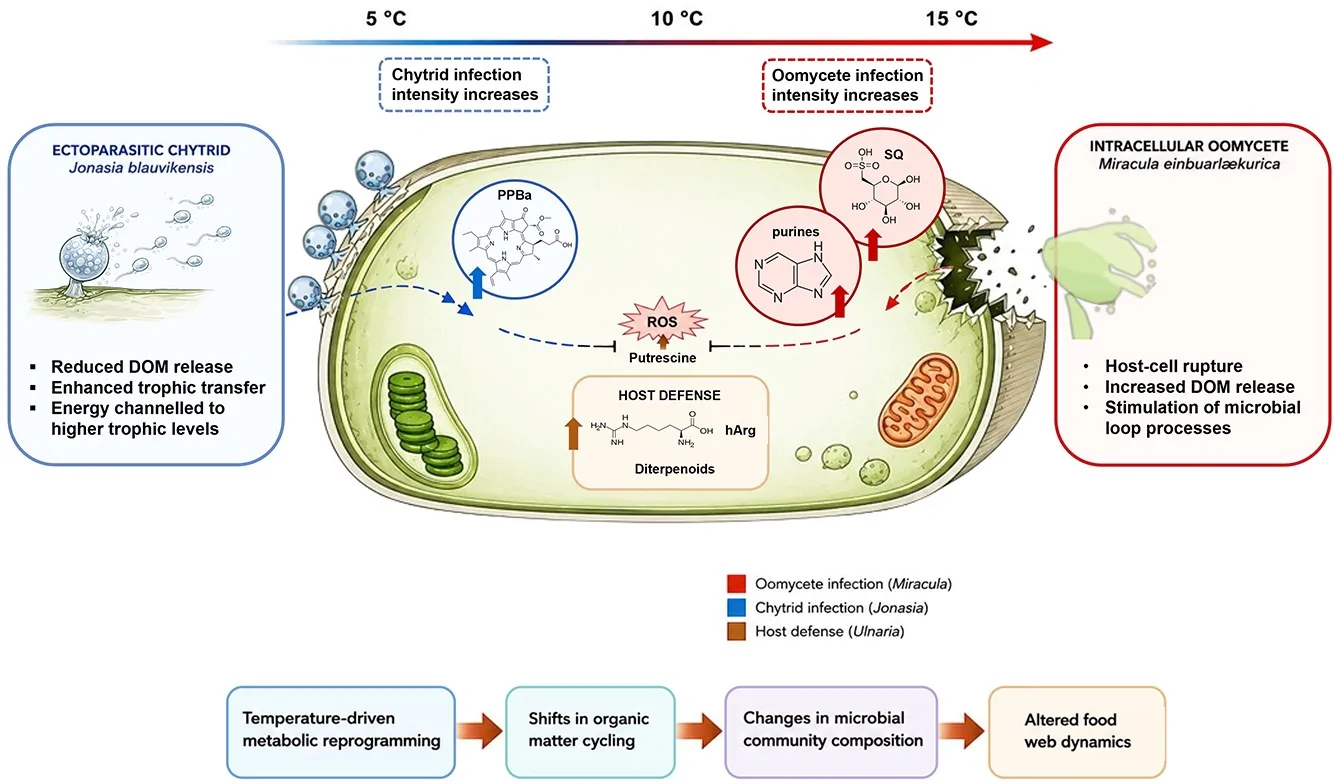
Conceptual overview of temperature- and infection-associated principal metabolites and hypothesized processes in the diatom *U. ulna* during interactions with chytrid (*Jb*) or oomycete (*Me*) parasites. A single diatom cell with key metabolic changes associated with chytrid (blue arrows) and oomycete (red arrows) infections. Brown arrow indicates host defense-related metabolic responses. Solid arrows represent metabolite associations supported by this study, whereas dashed arrows indicate hypothesized metabolic or ecological processes. Pheophorbide *a* (PPBa), detected specifically in chytrid-infected treatments, may reflect phaeoplast degradation processes during infection, and is potentially taken up by chytrids but released as DOM during oomycete infections. Putrescine and diterpenoid derivatives are associated with infected treatments and may be linked to parasites’ strategy to counteract host ROS defense. Unlike chytrid, oomycetes may scavenge sulfoquinovose (SQ) and purine from the host, reflecting different infection strategies between the parasites. Homoarginine (hArg) and diterpenoid derivatives may represent the host’s defense-related metabolic responses to parasite infection and are upregulated regardless of temperature (brown arrow). Potential ecological implications of temperature-associated metabolomic shifts, including effects on dissolved organic matter processing and aquatic food-web interactions, remain hypotheses requiring further investigation.

Additionally, pheophorbide *a*, a product of chlorophyll breakdown, was only detected in extracts of chytrid-infected cells, indicating cell lysis and oxidation of the central magnesium atom of intact chlorophyll (Fig. 3a). Sulfoquinovose was the only metabolite specific to oomycete infections (Fig. 3c), potentially indicating differences in nutrient acquisition strategies between oomycetes and chytrids. Some long-chain fatty acid, prostaglandin, and diterpenoid derivatives were also identified as significant features associated specifically with parasite treatment at 10°C and 15°C during analysis with the RP18 column (Supplementary Figs S1 and S2). A schematic overview of metabolites associated with chytrid and oomycete infections, and potential stress- or defense-related responses, is provided in Fig. 4.

## Discussion

Understanding host–parasite interactions, particularly those involving zoosporic parasites infecting microalgae and playing a key role in the demise of algal blooms, has recently received increased attention [3, 11, 40–42]. Here, we identify substantial shifts in the cellular metabolome of a recently introduced algal model system from the Arctic region in interaction with chytrids and oomycetes during temperature changes. Specifically, we investigated the interactions between the widespread benthic diatom *U. ulna* and two of its obligate biotrophic parasites, the chytrid fungus *J. blauvikensis* and the oomycete *M. einbuarlaekurica*, at the metabolomic level.

### Effect of temperature on diatom growth and infection prevalence

Diatom growth rate was measured as an increase in cell abundance over time. While a similar growth rate was observed at 10°C (optimal incubation temperature) and 15°C, the diatom host exhibited significantly slower growth at 5°C. Temperature plays a key role in regulating the rate of metabolic processes, which directly influences algal growth (Q10) [43]. While different diatom species may respond differently to temperature changes [44], diatom division rates tend to rise with increasing temperature, peaking at an optimal range before declining as the temperature moves away from this optimum [45]. Our results are in line with other laboratory-based studies [46, 47], which tested several diatom species from an Arctic fjord (Kongsfjorden, Svalbard) and showed that their optimum growth temperatures ranged from 12 to 15°C, yet they grew more slowly at lower temperatures. The same pattern has also been detected in natural, complex diatom populations from temperate regions [48]. Although no *in situ* temperature tolerance experiments have yet been conducted specifically for this strain, Salimi et al. [30] reported the occurrence of *Ulnaria* throughout the year under varying environmental conditions, including colder periods, while also observing slower growth at lower temperatures. Temperature is also a key factor shaping parasite–host interactions, with colder conditions often hypothesized to provide a refuge for phytoplankton hosts by limiting parasite development and transmission, a concept known as the cold water refuge theory [49]. However, whether this pattern applies broadly across different parasite groups and host–parasite systems remains unresolved. In the chytrid-infected treatment, *Ulnaria* exhibited significantly slower growth at lower temperatures. Because the number of infected cells was not significantly affected by temperature, the patterns observed suggest this chytrid appears to maintain infection success at low temperatures, consistent with the observations of Salimi et al. [30], who detected *J. blauvikensis* in all seasons, including winter. Chytrid infections were similarly effective across the tested temperature range (5–15°C), but the growth of the algal host was more strongly affected. Consequently, chytrid infection prevalence increased at lower temperatures, challenging the general applicability of the cold water refuge theory in this system [49], as low temperature did not substantially limit chytrid infectivity under our experimental conditions. Yet at higher temperatures, the chytrid showed rapid early infection dynamics, but the algal host subsequently outgrew the parasite. This short-term dynamic led to a decrease in chytrid infection prevalence by the final day of the experiment (Day 7). Under long-term culture conditions, chytrid infections can eventually overwhelm the host [30]. Additionally, in this study, we did not measure other parameters of chytrids, such as the number of zoospores per infected host cell, zoospore lifetime, or other factors affecting the transmission rate that are known to be influenced by temperature [50]. However, chytrid transmission rates are generally considered to be influenced primarily by host density [6]. As we observed increased chytrid infection prevalence at lower temperatures despite lower host cell abundance, it is important to note that the density impact on the transmission success occurs only when host density falls below a certain threshold (e.g. 170 to 500 cells ml^−1^ in the case of the diatom *Fragilaria crotonensis* or 10 to 100 cells ml^−1^ in the case of *Asterionella formosa* [51]). In our culture conditions, host cell abundance was consistently high enough; thus, density is not considered a limiting factor.

In the oomycete-infected (*Me*) treatment, the highest number of infected cells was observed at 15°C. At 5°C, the number of non-infected *Ulnaria* hosts declined over time, increasing overall infection prevalence, and again refuting the cold-refuge hypothesis [49]. This is in contrast to the chytrid-infected (*Jb*) treatment, in which the non-infected host population continued to grow throughout the experiment, although at a slower rate. Differences in these parasite infection patterns may be explained by the distinct infection mechanisms used by oomycetes. For example, Vallet et al. [17] demonstrated that the oomycete *L. coscinodisci* can directly manipulate the host’s metabolome to maximize its multiplication. This involves compounds that arrest algal cell division, thereby limiting host proliferation and optimizing the oomycete’s access to host cell content and energy for its multiplication. In contrast, chytrids may employ strategies that avoid cell-cycle arrest in non-infected hosts, thereby ensuring a higher host cell density for transmission. In the model system used in this study, multiple chytrid infections were observed on a single *Ulnaria* cell, in line with Salimi et al. [30], who reported more than 50 sporangia per infected host cell. One possible explanation is that chytrids require fewer nutrients to grow into a small but mature thallus and prioritize fast growth and reproduction.

### Temperature affects the cell metabolome of the diatom *Ulnaria* during interaction with chytrid and oomycete parasites

The metabolomic dataset represents an integrated endpoint of infection-associated metabolic states rather than a time-resolved trajectory of metabolic change, reflecting an integrated signal across asynchronous infection stages. The observed metabolite patterns likely reflect a combination of processes occurring throughout infection progression, including host responses, parasite development, and late-stage physiological decline. Accordingly, metabolic profiles of extracts from *Ulnaria* (non-infected), chytrid- and oomycete-infected cells were clearly distinguishable regardless of temperature conditions, indicating pronounced infection-associated changes in the host metabolome. These differences are consistent with the contrasting infection strategies of the two parasite groups. Oomycetes are endoparasites that grow within the host cell and eventually cause its rupture. In contrast, chytrids are ectoparasites that develop externally on the host surface, extracting nutrients while leaving the cell intact. Such distinct modes of nutrient acquisition may help explain the divergent metabolomic signatures observed in our study. Infected diatom treatments showed elevated signals for several specific metabolites, suggesting either their parasite origin or *de novo* production by the host in response to parasite infection. Among these, homoarginine may be associated with defense-related responses. Specifically, L-homoarginine disrupts phosphorus metabolism in fungal hyphae and generally acts as an antifungal compound [52]. The highest concentration of homoarginine was detected in the chytrid-infected treatment at 15°C, consistent with microscopy observations that revealed a lower chytrid infection prevalence at this temperature. This observation suggests that hosts are more effective at defending themselves at 15°C than at 5°C, where homoarginine was completely absent and chytrid infection prevalence was higher. We propose that this may be due to reduced host metabolic rates at lower temperatures.

In contrast, other metabolites with elevated signals in infected treatments appear to originate from the parasites themselves. For instance, phosphatidylcholine (PC) and phosphatidylethanolamine (PE) are the most abundant phospholipids in eukaryotic cells, including yeast and fungi, and perform crucial functions in the maintenance of membrane integrity [53] and play key roles in virulence of the fungus *Candida albicans* [54]. The amphibian-infecting chytrid *Batrachochytrium dendrobatidis* produces significant amounts of the polyamine spermidine [55], derived from putrescine. Although we did not detect spermidine in our study, which may be due to limitations in our extraction method, we identified putrescine across all treatments (Fig. 2). This finding is consistent with its role as a ubiquitous polyamine, essential for growth, cell division, and environmental stress tolerance in algal species [56, 57]. Putrescine also plays a significant role in reactive oxygen species (ROS) tolerance by scavenging ROS and enhancing antioxidant defense capacity, or by inhibiting ROS generation [58]. The presence of putrescine in non-infected treatments aligns with its fundamental cellular functions, but its levels were significantly elevated in infected treatments. The elevated putrescine levels may reflect activation of pathways associated with oxidative stress responses, which provide a defense against ROS generated by the host cells during the infection. This strategy would allow them to avoid oxidative damage and maintain their infection process. However, further studies are needed to clarify its exact function in host–parasite interactions in aquatic environments.

In host–parasite interactions, signaling, and overall pathogenicity, oxylipins play a crucial role [59]. In our study, we identified several oxylipins in infected diatom treatments at 10 and 15°C, including prostaglandin and diterpenoid derivatives. Prostaglandins are essential factors in promoting fungal colonization, chronic infection [60], and have been linked with growth regulation and sexual maturation in the oomycete parasite *Lagenidium giganteum* [61]. Prostaglandins detected in our study may originate from the parasites themselves. However, further studies are needed to determine their specific roles in diatom–parasite interactions. Moreover, the detected diterpenoids may originate from the host, as diterpenoids are often considered fungicides with significant antifungal activity [62].

The detection of some metabolites was linked explicitly to chytrid infections and was not associated with oomycete infections, including chaetomellic acid and pheophorbide *a*. Chaetomellic acid has previously been shown to interfere with membrane-associated signaling pathways in eukaryotic cells [63, 64]. In the context of chytrid infection, this interference may potentially influence the host’s essential cellular functions, weaken its defenses, and dominate the host’s cellular environment. Pheophorbide *a* is a product of chlorophyll degradation [65] and a signal molecule capable of inhibiting specific enzymes and inducing cell death [66]. Our study detected the highest concentration of pheophorbide *a* in chytrid-infected treatments at 5°C, where infection prevalence peaked on the final day of the experiment. However, it remains unclear why pheophorbide *a* was absent in all oomycete treatments, despite reports by Vallet et al. [18] of high pheophorbide *a* concentrations in their study on the oomycete *L. coscinodisci* infecting the diatom host *Coscinodiscus* spp. Different oomycete species may degrade chlorophyll pigments differently, depending on the diatom host.

Sulfoquinovose was among the specific metabolites associated with oomycete-infected cells. We hypothesize that this compound may be a degradation product produced or utilized by the oomycete during growth within the algal cell. Similar to pathogenic bacteria like *Pseudomonas*, which synthesize membranous glycolipids as an alternative to phospholipids under low-phosphate conditions [67], the oomycete may exploit sulfoquinovose via the SQ-degradation pathway to support its growth. This observation aligns well with our cell count data, showing the highest number of infected cells and the highest sulfoquinovose concentration at 15°C. Furthermore, the detection of a purine derivative specific to oomycete-infected cells, with the highest abundance at elevated temperature where infection prevalence peaked, suggests a role for the purine salvage pathway in *Miracula*–*Ulnaria* interactions. Some parasitic protists cannot synthesize purines *de novo* and instead rely on salvaging purines from their hosts for survival [68]. This finding aligns with the hypothesis of Vallet et al. [18] that the oomycete leverages purine salvage mechanisms during host invasion and cellular breakdown. Nevertheless, it remains an open question which of the detected metabolites are involved in the oomycete infection mechanism under low-temperature conditions. A decreased number of non-infected host cells and a relatively high number of infected cells might suggest that the oomycete utilizes mechanisms that prevent the host from reproducing. Alternatively, signaling molecules produced by the algal host during infection may trigger the host’s self-destruction, thereby limiting pathogen spread.

Beyond shaping direct host–parasite interactions, temperature-associated changes in the metabolome of infected *Ulnaria* cells may influence the processing of organic matter in aquatic ecosystems. The observed temperature-driven differences in the types and quantities of metabolites released upon infection could potentially influence microbial community composition, as distinct metabolites provide distinct substrates and signaling cues for other organisms. Although these ecological implications were not directly measured in the present study, they are supported by previous reports describing functional roles of several detected compounds in other host–microbe interactions. One noteworthy observation from our experiment is that the two parasites appear to induce distinct infection outcomes. Oomycetes cause rupture of the host cell, potentially releasing substantial dissolved organic matter (DOM) that could potentially stimulate the microbial loop processes, whereas chytrid infections do not lyse the host cell and may therefore contribute less to DOM fluxes. These differences and temperature-mediated outcomes may ultimately cascade to higher trophic levels, highlighting possible connections between cell-level infection processes and broader food web dynamics that warrant further investigation.

## Supplementary Material

Ilicic_et_al_2026_Supplementary_Material_ycag240

## Data Availability

The microbial strains are maintained continuously and available upon request from M.T. The datasets are available at MassIVE spectral database under Accession number MSV000097103. The identification using ddMS acquisition on the QC pool samples and analysis with molecular networking in GNPS can be found under Accession number MSV000097102.
