# Supplementary material for: Temperature modulates infection-associated metabolic responses in bloom-forming freshwater diatoms
Source: ISME Commun. 2026 Aug 26;6(1):ycag240. doi: 10.1093/ismeco/ycag240 (PMC13584213; doi:10.1093/ismeco/ycag240)
Supplement: Supplementary file 1 [file ilicic_et_al_2026_supplementary_material_ycag240.pdf]

Supplementary materials

Temperature modulates infection-associated metabolic responses in bloom-forming freshwater diatoms

Doris Ilicic[+] [1], Marine Vallet[+] [2,3], Fatemeh Salimi [4,5], Anthony T. Buaya [4], Marco Thines [4,5*], Hans-Peter Grossart [1,6*]

[1] Department of Plankton and Microbial Ecology, Leibniz Institute of Freshwater Ecology and Inland Fisheries, Neuglobsow, Germany
[2] Institute for Inorganic and Analytical Chemistry, Friedrich Schiller University Jena, Jena, Germany
[3] Max Planck Fellow Group Plankton Community Interaction, Max Planck Institute for Chemical Ecology, Jena, Germany
[4] Senckenberg Biodiversity and Climate Research Centre, Frankfurt (Main), Germany
[5] Department of Biological Sciences, Institute for Ecology, Evolution and Diversity, Goethe University, Frankfurt (Main), Germany
[6] Institute of Biochemistry and Biology, University of Potsdam, Potsdam, Germany

[+] Shared first co-authorship
* Correspondance: hgrossart@igb-berlin.de, m.thines@thines-lab.eu

Supplementary Figures

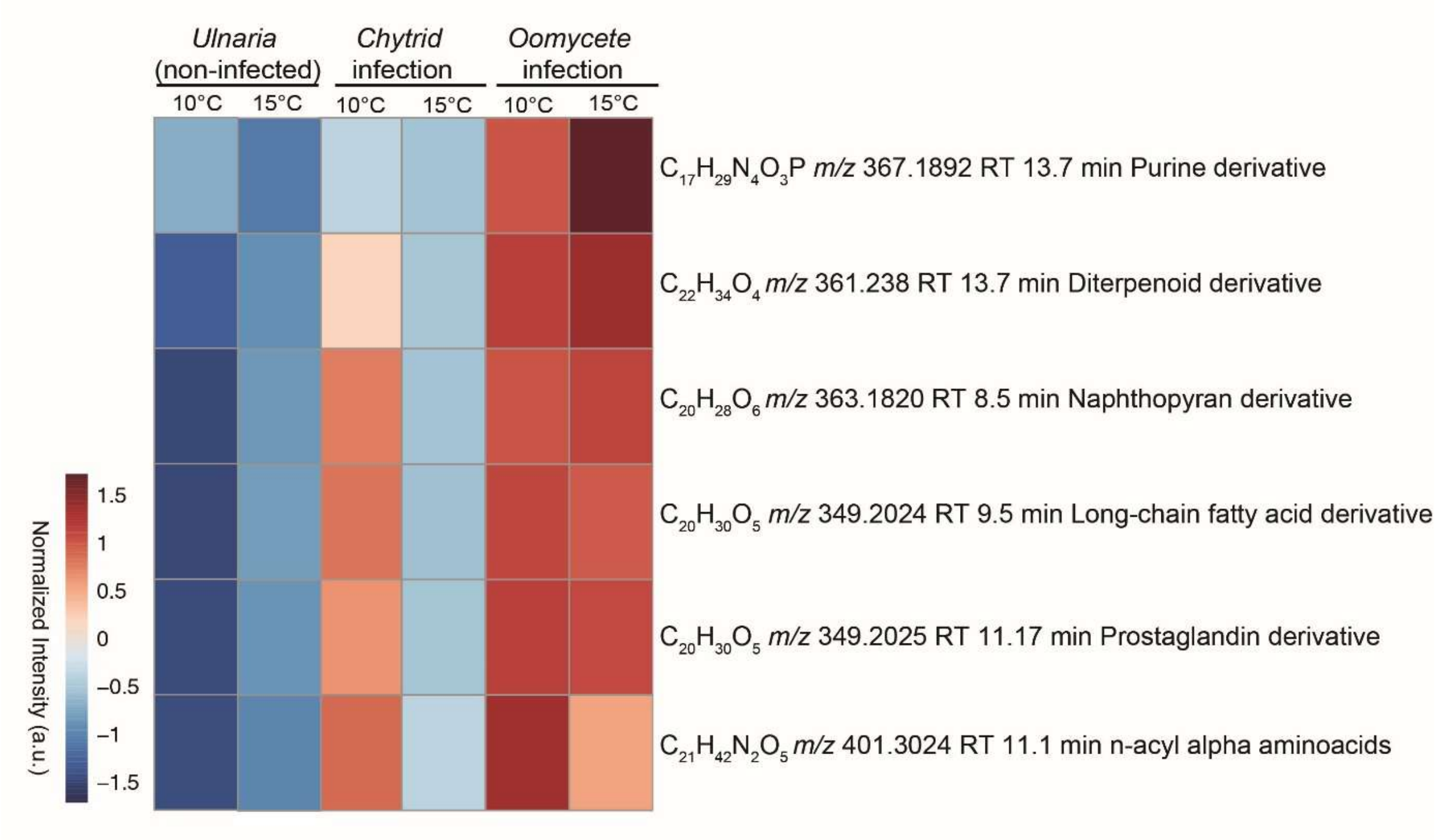

Figure S1. Identification of the significant metabolites associated with chytrid and oomycete infection in *Ulnaria ulna* cell extracts using LCMS analysis with a RP18 column. Intensities of the peak areas were normalized based on cell count, log-transformed, and Pareto-scaled.

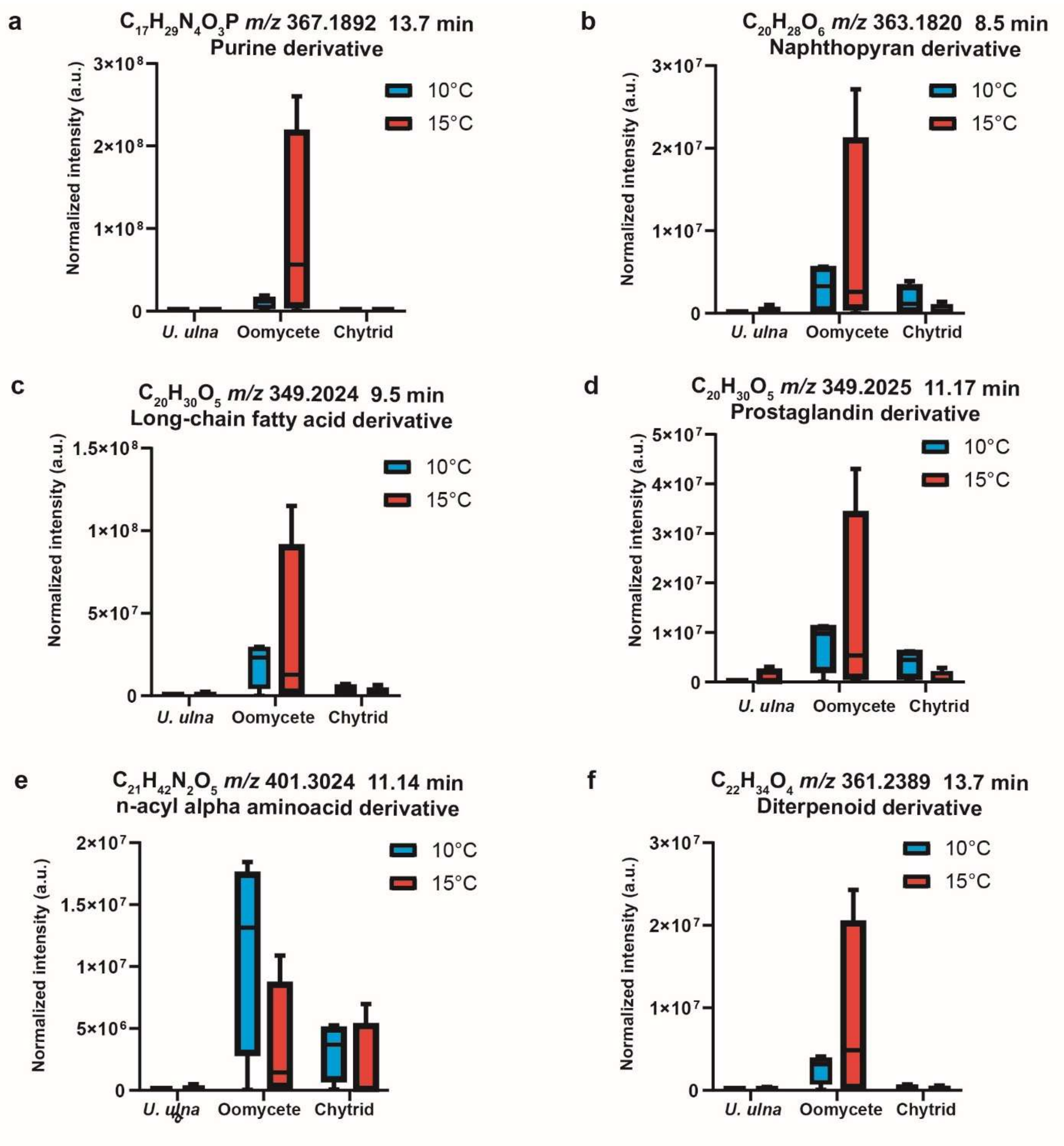

Figure S2. Abundance of metabolites significantly up-regulated detected using RP18 column chromatography during parasite infection at different incubation temperature (10°C and 15°C). The identification is based on a spectral similarity matching of MS2 fragmentation with MS2 spectra from the public database in GNPS and fragmentation analysis was done with SIRIUS CSI:FingerID. Intensities of peak areas were normalized based on cell counts, log-transformed, and Pareto-scaled.

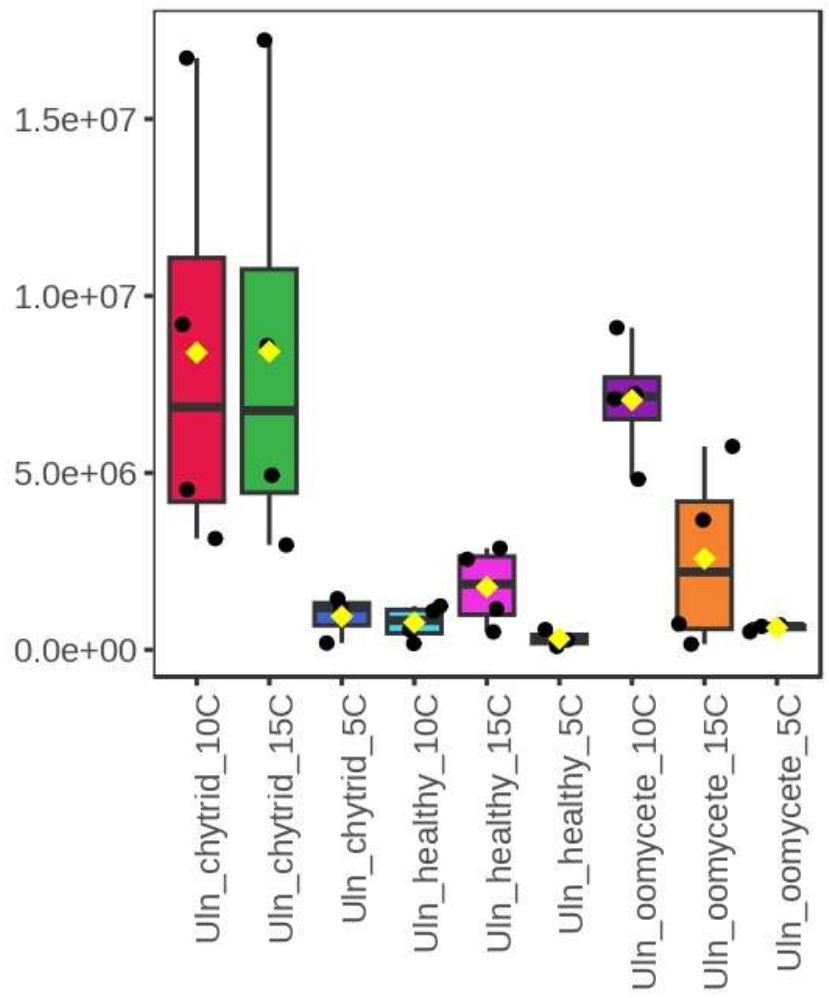

Figure S3. Abundance of the feature $C_4H_{12}N_2$ (retention time 7.2 min, *m/z* 89.1038) assigned as putrescine (putative annotation, MSI level 2) that was found significantly up-regulated during parasite infection, regardless of the temperature. The identification is based on a spectral similarity matching of MS2 fragmentation with MS2 spectra from public databases. Intensities of the peak areas were normalized based on cell count, log-transformed, and Pareto-scaled.

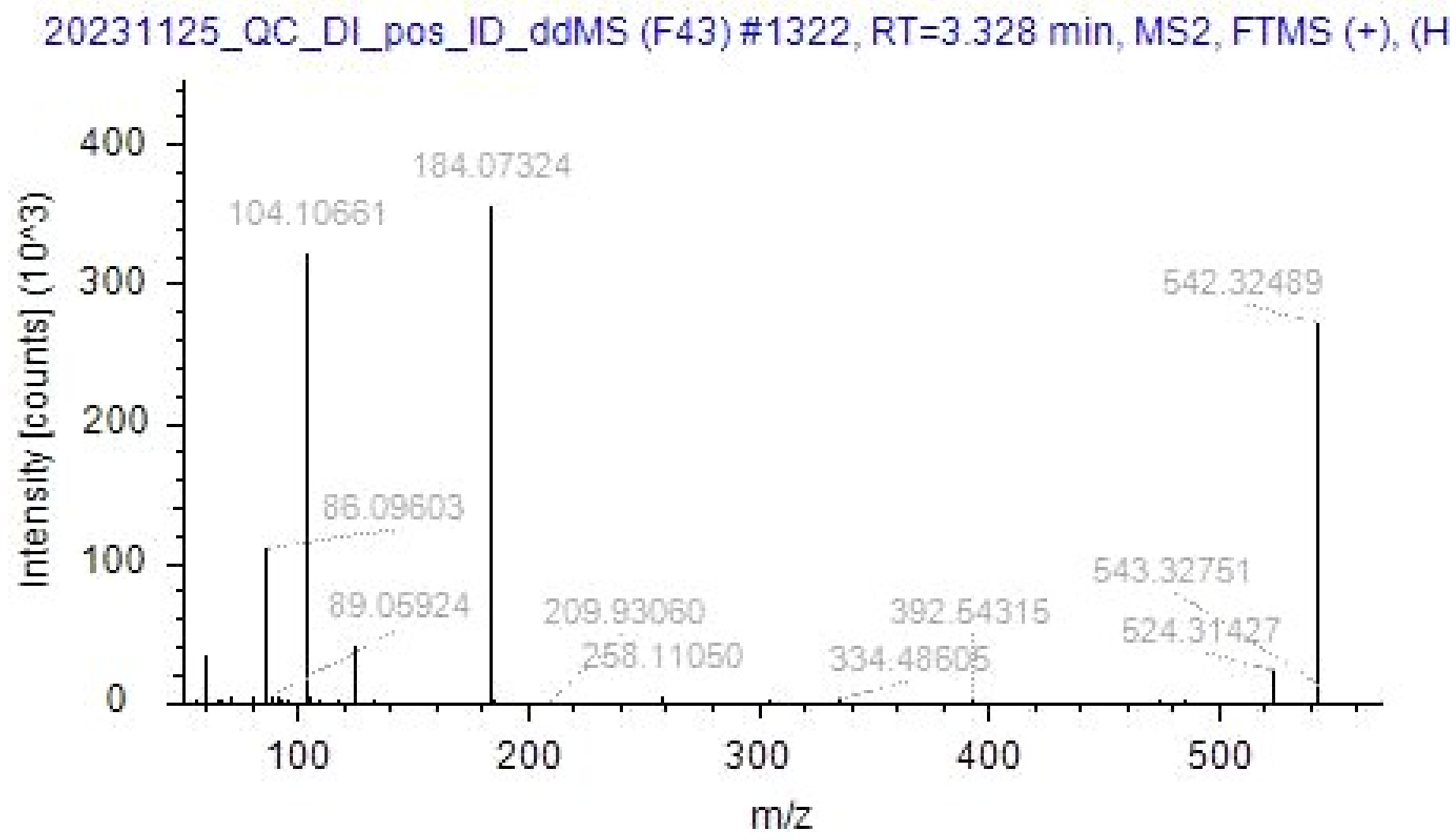

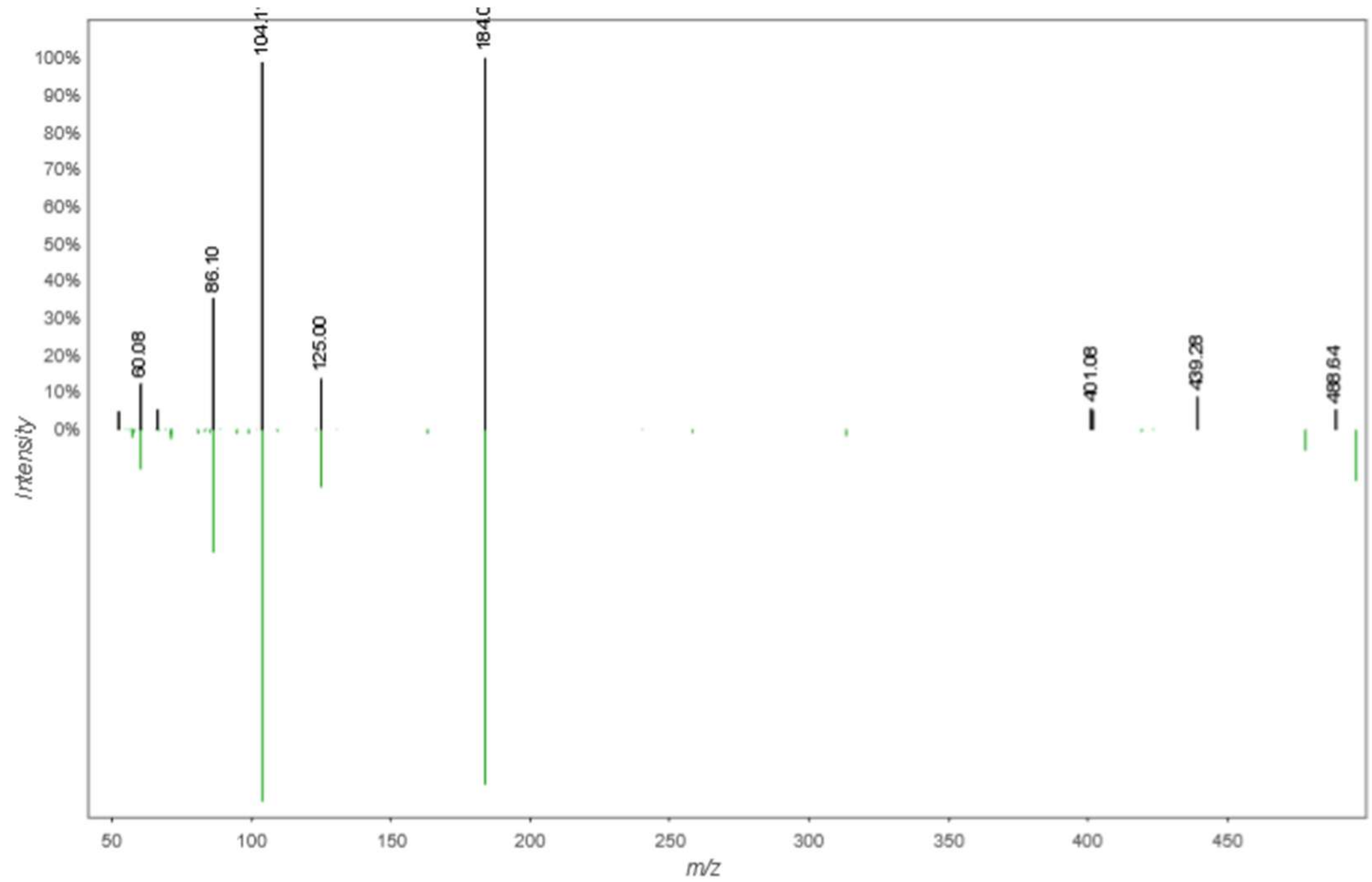

Figure S4. MS/MS spectrum for the feature $C_{28}H_{48}NO_7P$ *m/z* 542.32 RT 3.3 min assigned to PC(20:5/0:0). (ZIC-HILIC dataset). Spectral Match to Lyso-PC(16:0) from NIST14 cosine score 0.90. https://gnps.ucsd.edu/ProteoSAFe/gnpslibraryspectrum.jsp?SpectrumID=CCMSLIB00003135346#%7B%7D

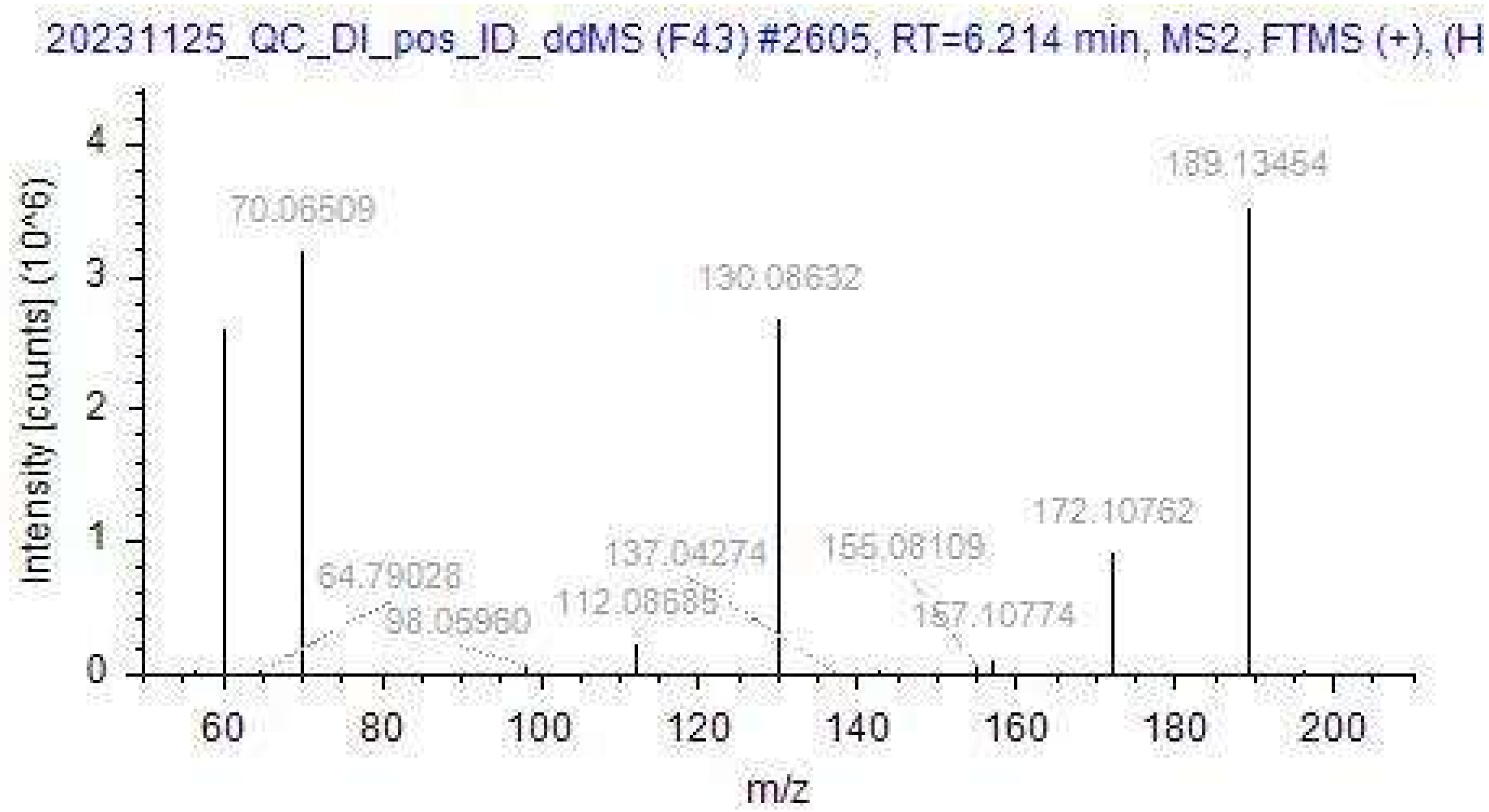

Figure S5. MS/MS spectrum for the feature $C_7H_{16}N_4O_2$ *m/z* 189.13 RT 6.2 min assigned to homoarginine (ZIC-HILIC dataset).

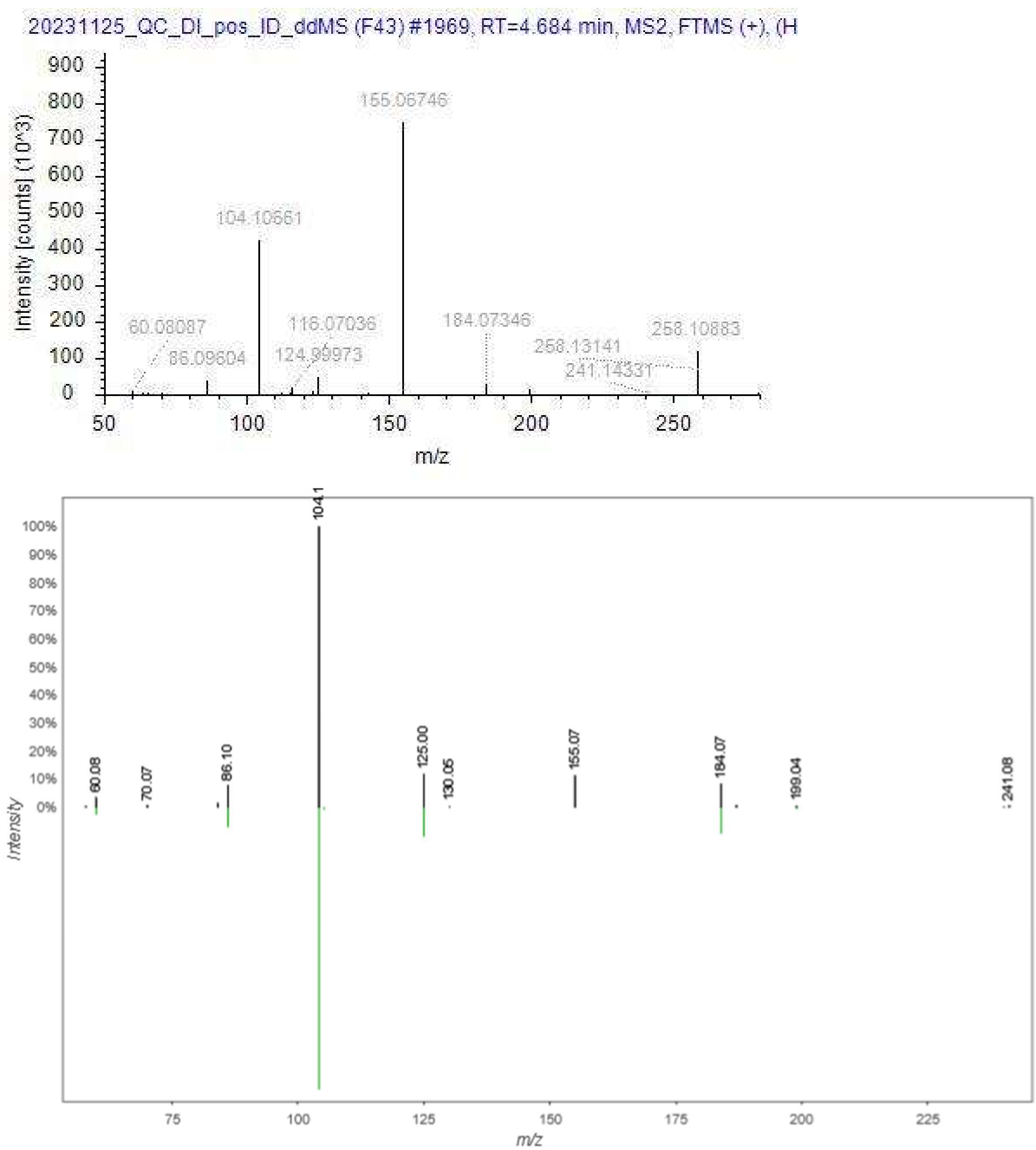

Figure S6. MS/MS spectrum for the feature $C_8H_{20}NO_6P$ *m/z* 258.10 RT 4.6 min assigned to Choline alfoscerate/sn-glycero-3-phosphocholine (ZIC-HILIC dataset). match with that molecule with cosine score of 0.94.
https://gnps.ucsd.edu/ProteoSAFe/gnpslibraryspectrum.jsp?SpectrumID=CCMSLIB00006679241#%7B%7D

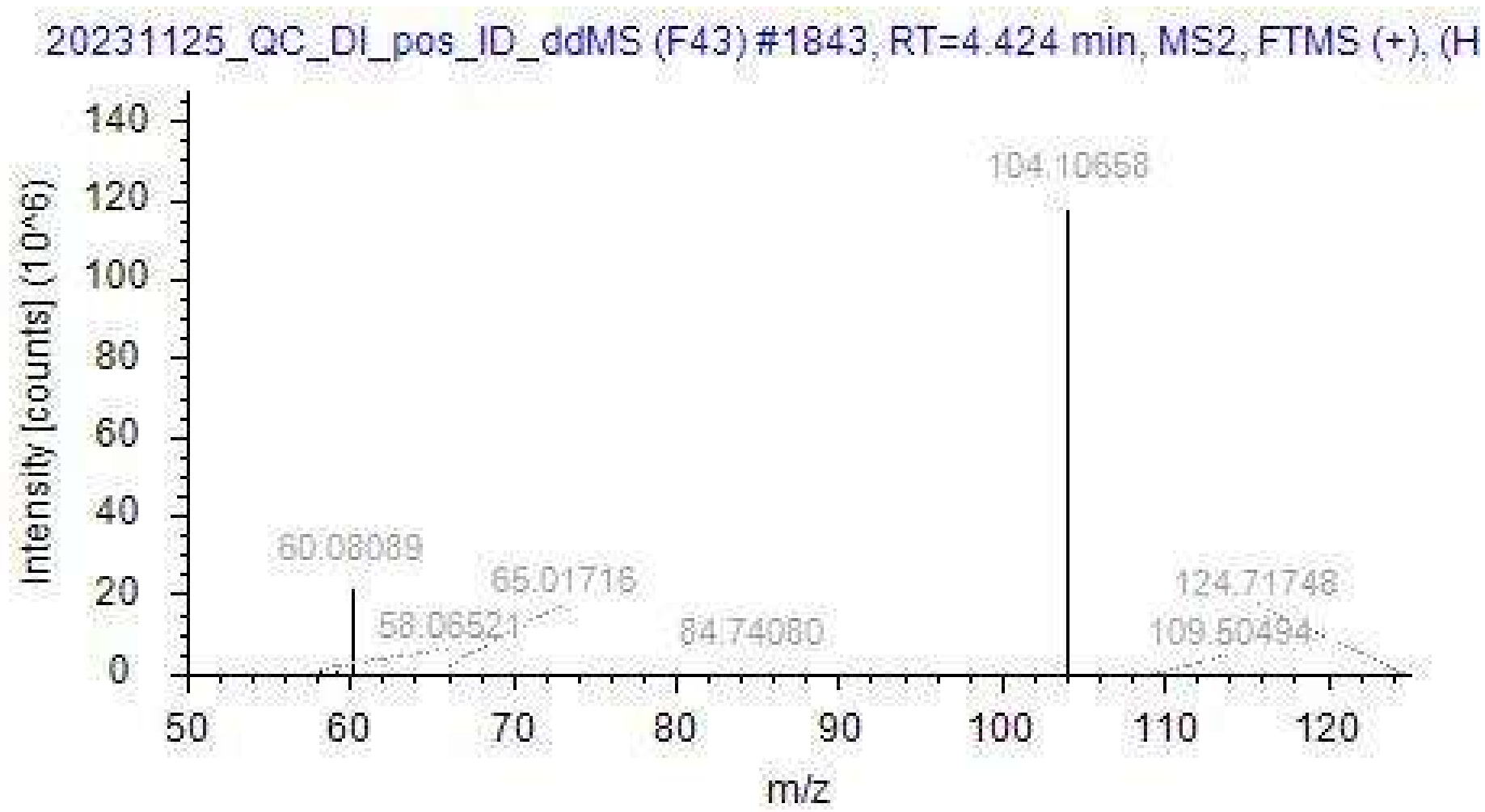

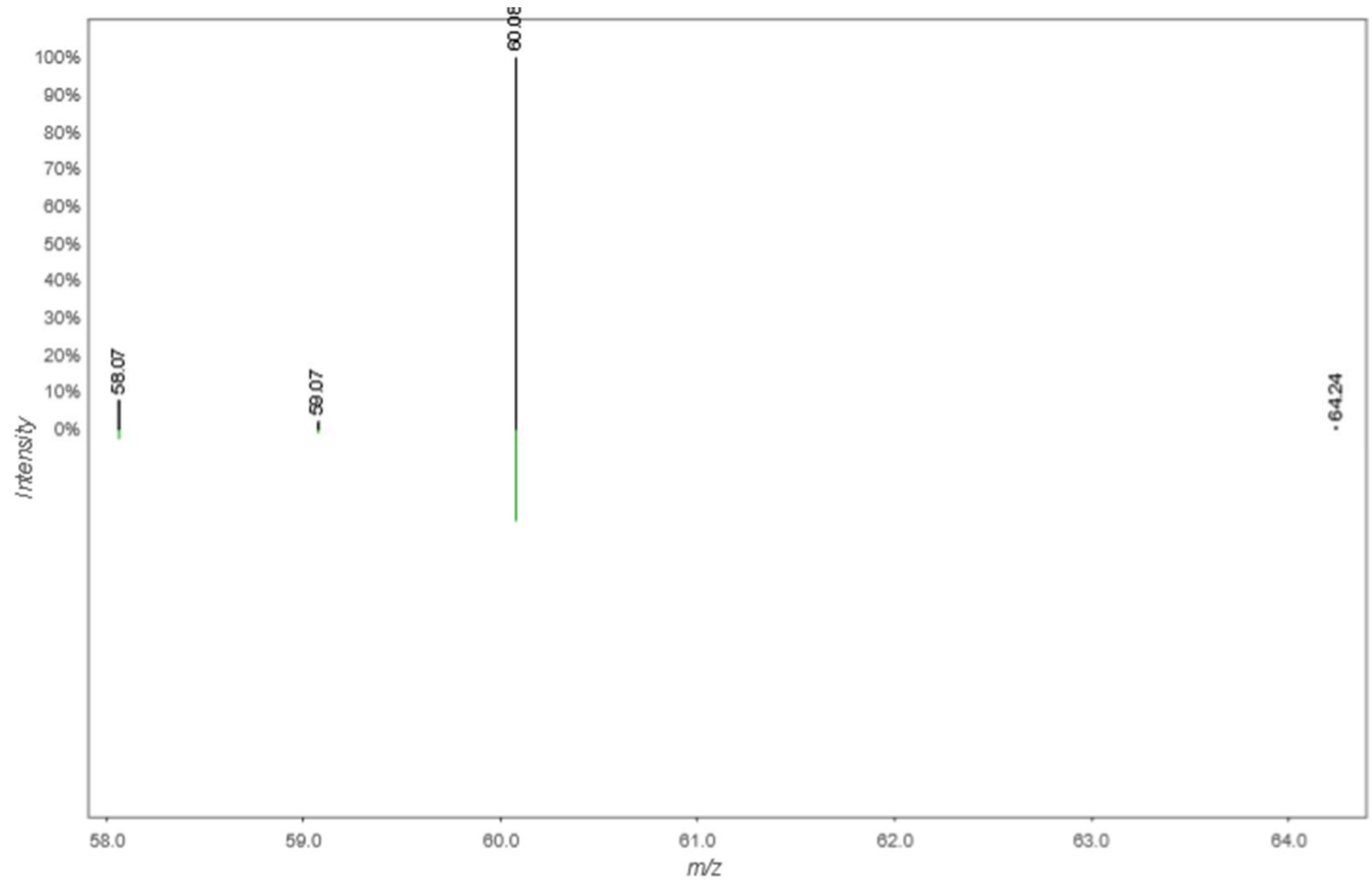

Figure S7. MS/MS spectrum for the feature $C_5H_{13}NO$ *m/z* 104.10 RT 4.4 min assigned to Choline (ZIC-HILIC dataset). Choline match with cosine score of 0.99.
https://gnps.ucsd.edu/ProteoSAFe/gnpslibraryspectrum.jsp?SpectrumID=CCMSLIB00006678341#%7B%7D

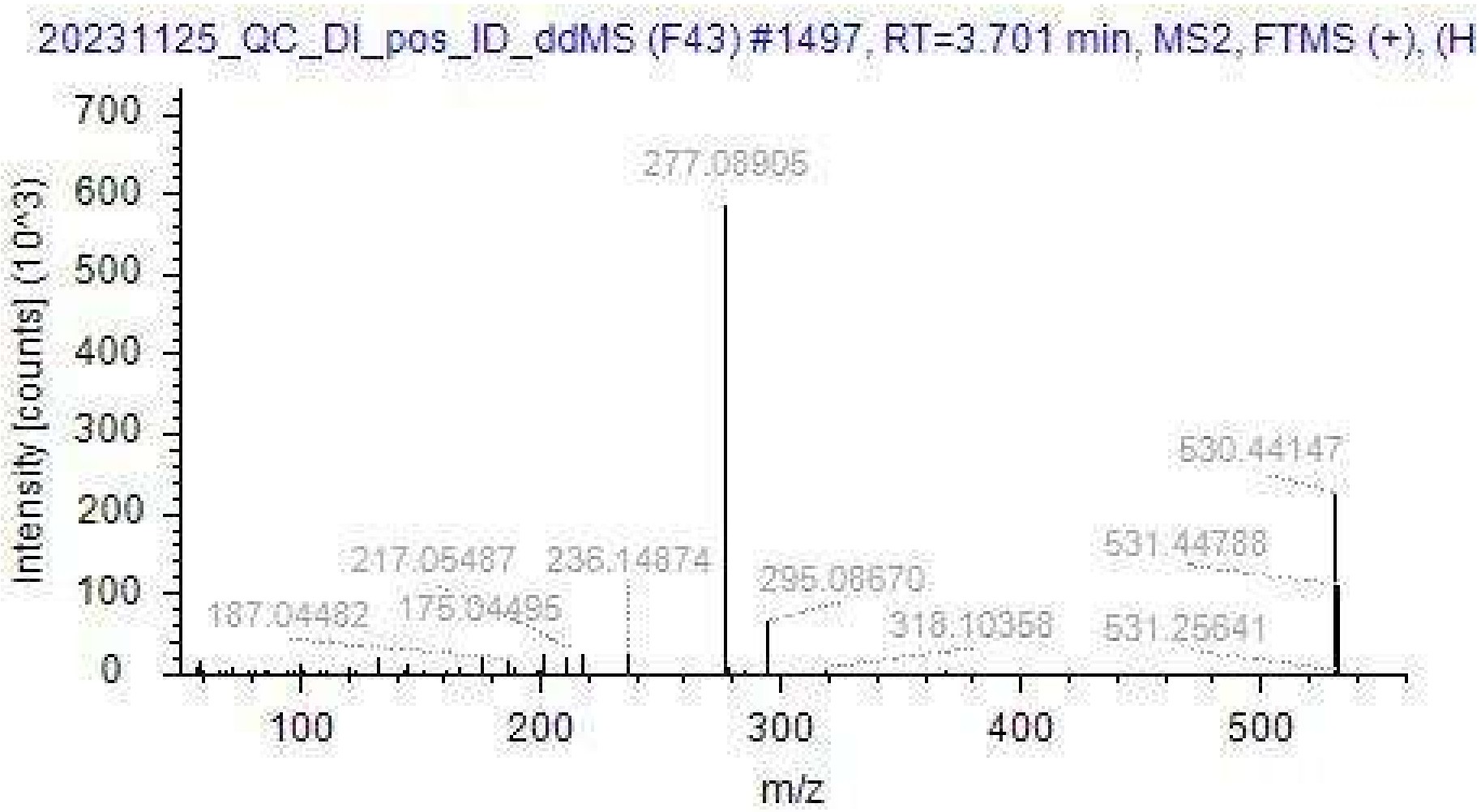

Figure S8. MS/MS spectrum for the feature $C_{26}H_{55}N_7O_4$ *m/z* 530.44 RT 3.7 min assigned to aminoacid derivative metabolite (ZIC-HILIC dataset).

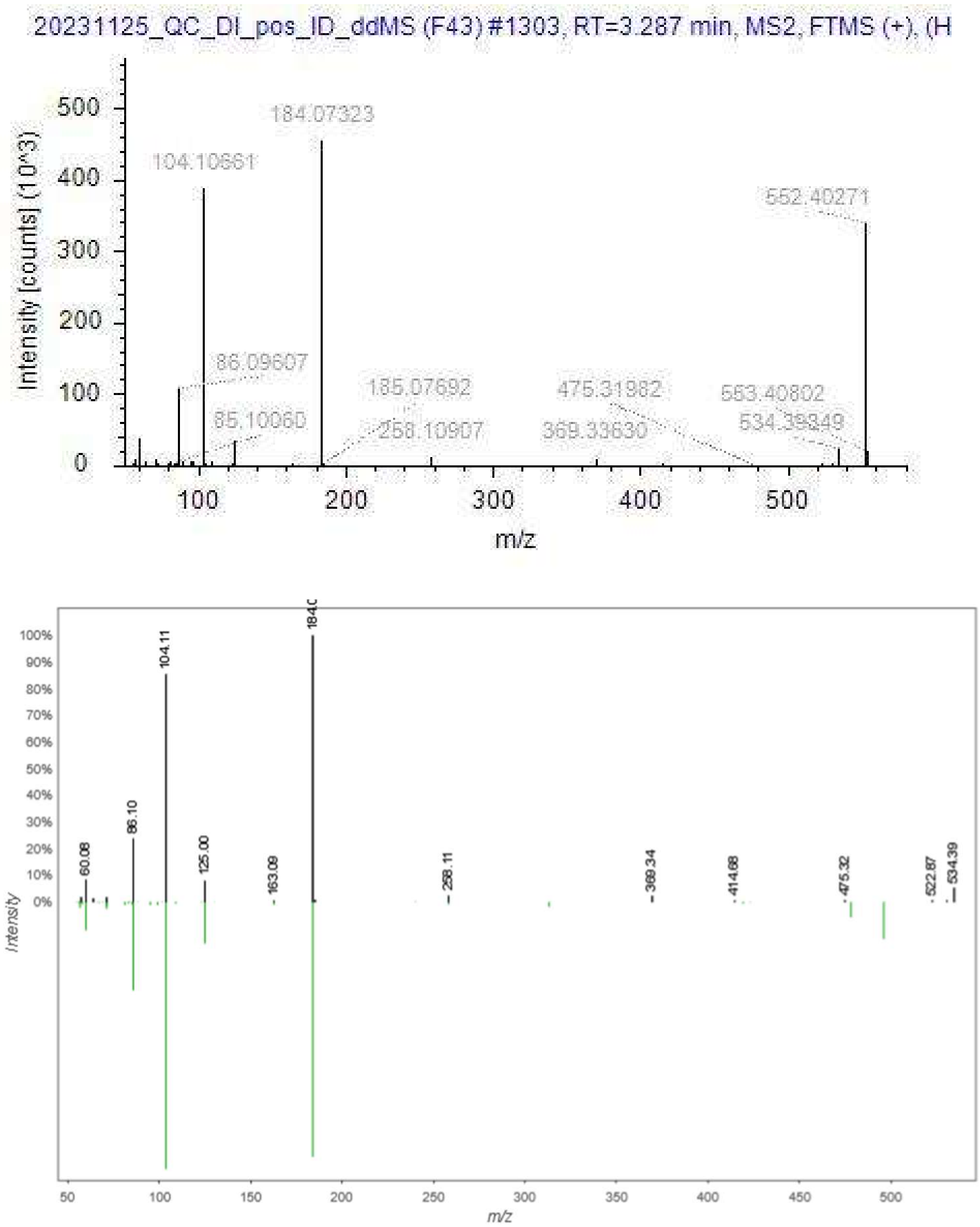

Figure S9. MS/MS spectrum for the feature $C_{28}H_{58}NO_7P$ *m/z* 552.40 RT 3.2 min assigned to LysoPC (20:0) (ZIC-HILIC dataset). Match to Lyso-PC (16:0) from NIST14 with cosine score of 0.98. https://gnps.ucsd.edu/ProteoSAFe/gnpslibraryspectrum.jsp?SpectrumID=CCMSLIB00003135346#%7B%7D

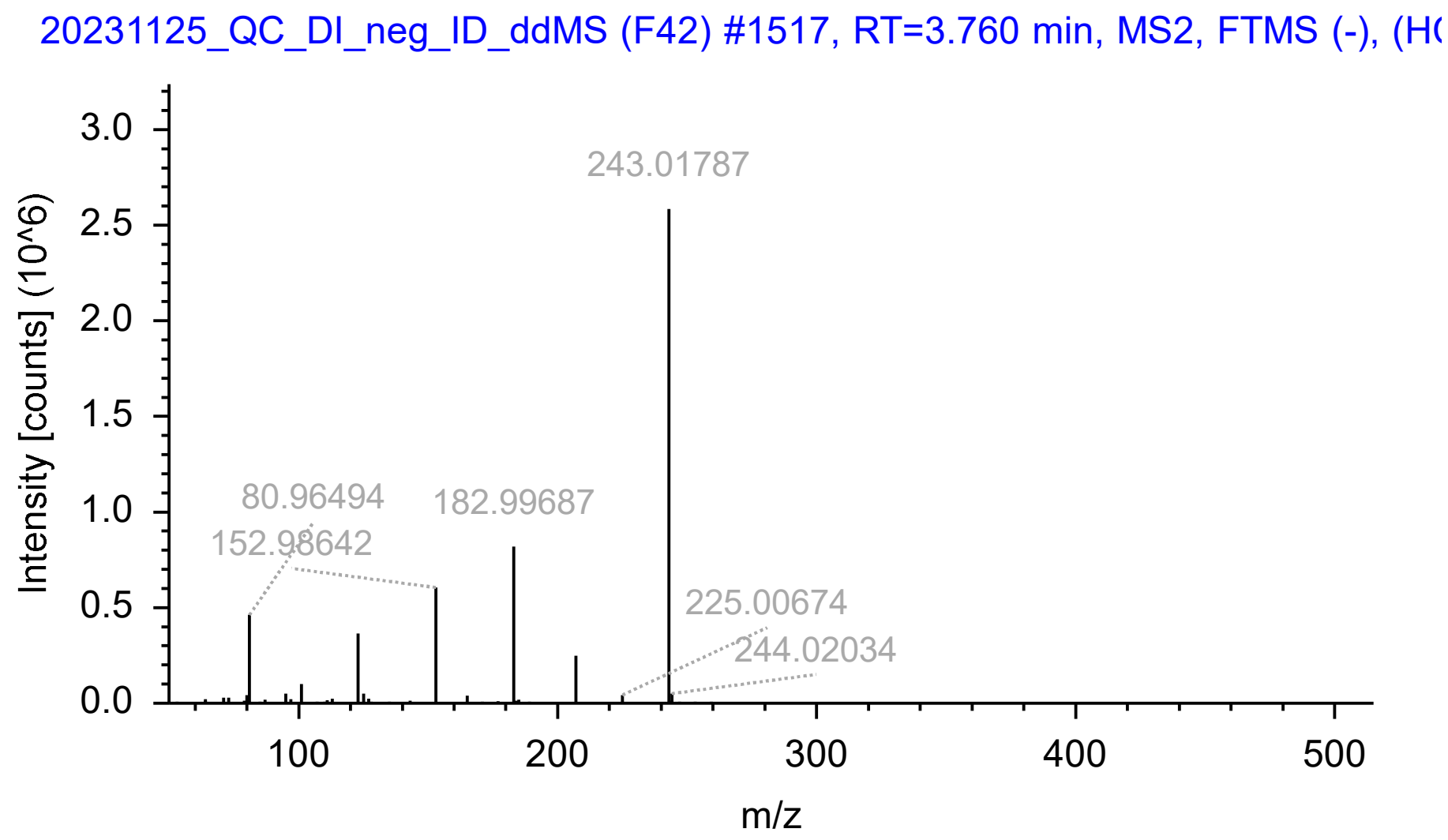

Figure S10. MS/MS spectrum for the feature $C_6H_{12}O_8S$ *m/z* 243.01 RT 3.7 min assigned to sulfoquinovose (ZIC-HILIC dataset).

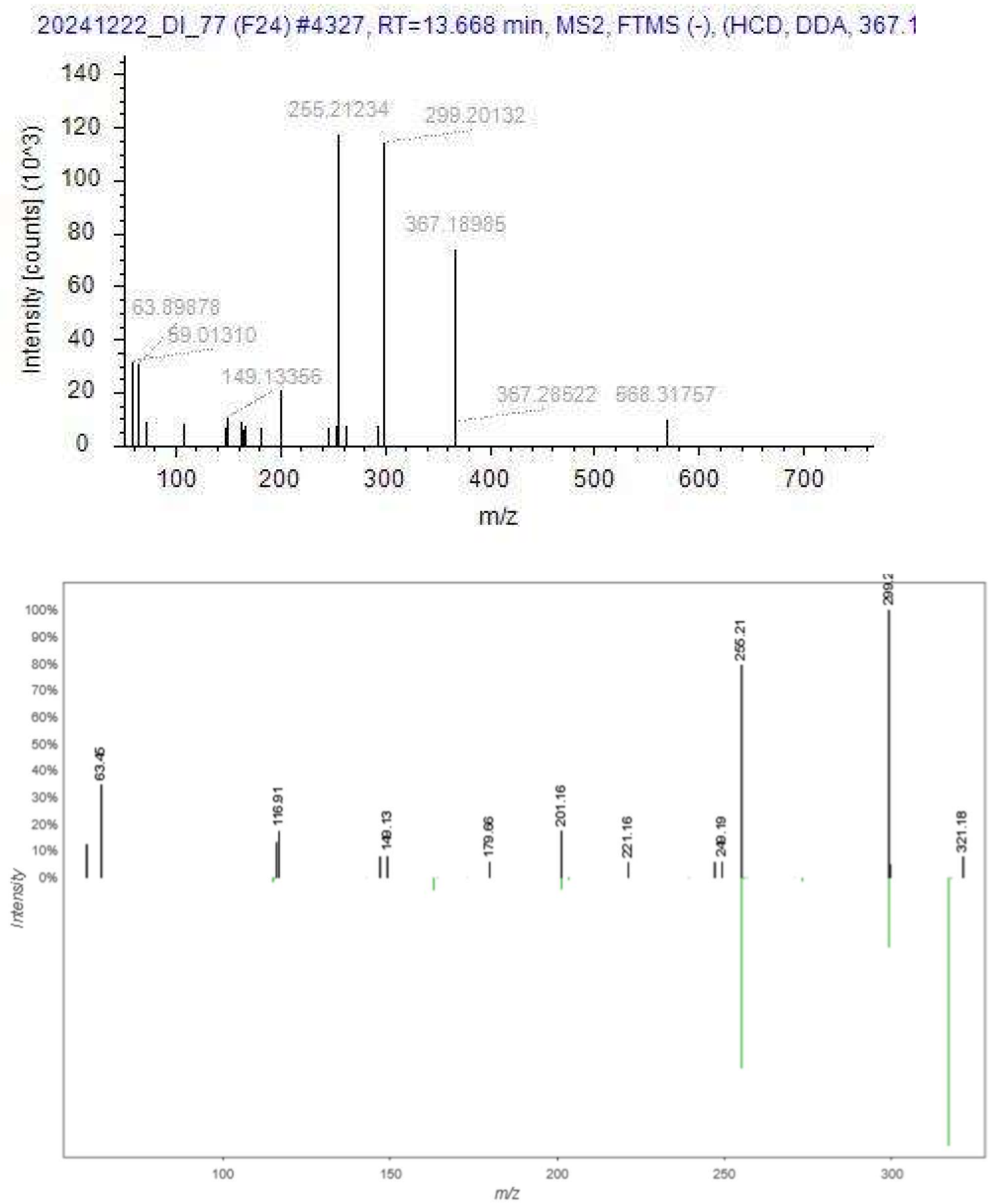

Figure S11. MS/MS spectrum for the feature $C_{17}H_{29}N_4O_3P$ *m/z* 367.18 RT 13.7 min assigned to purine derivative metabolite (RP18 dataset). match to 5-HEPE with cosine score of 0.71. https://gnps.ucsd.edu/ProteoSAFe/gnpslibraryspectrum.jsp?SpectrumID=CCMSLIB00005752139#%7B%7D

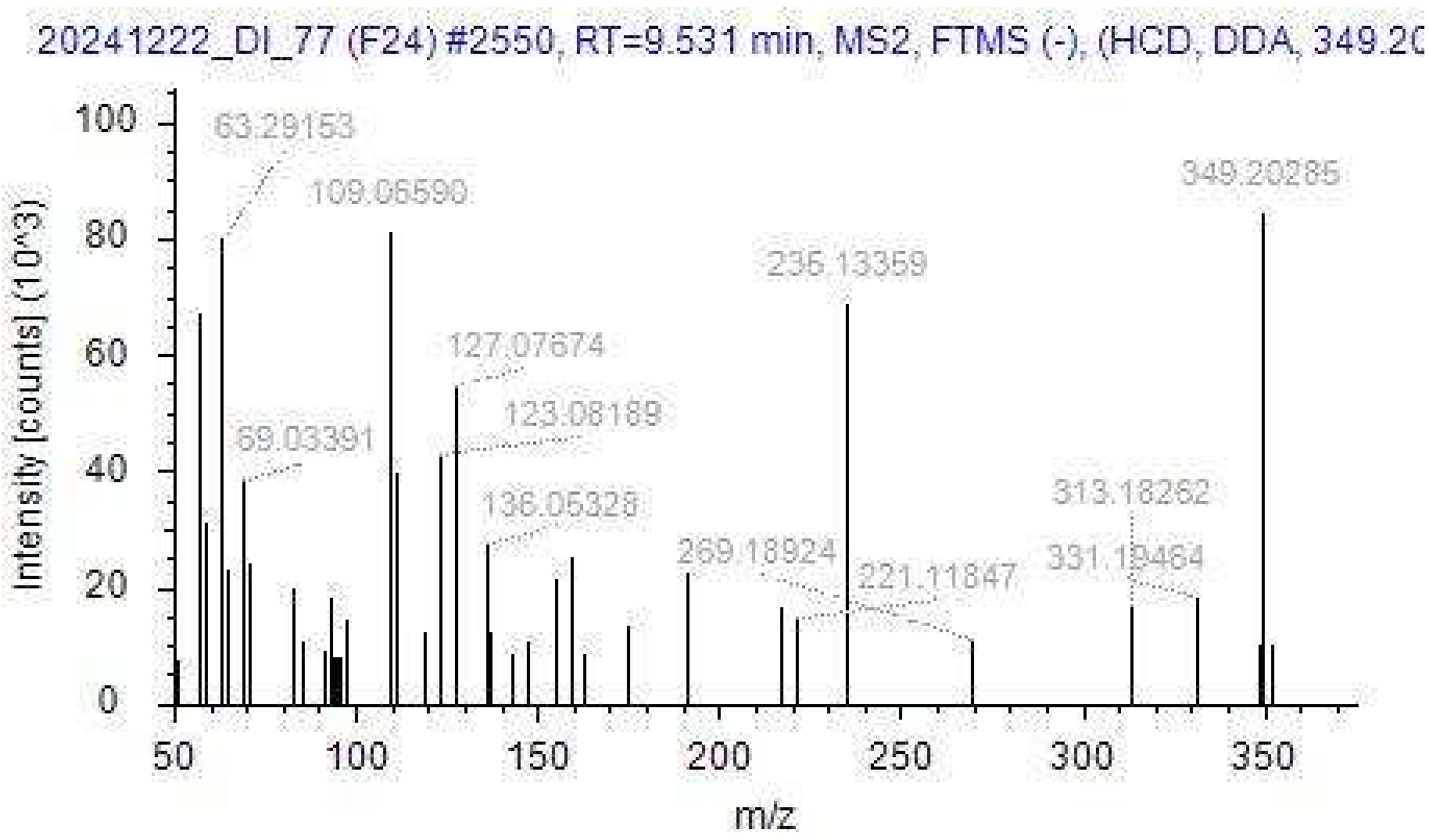

Figure S12. MS/MS spectrum for the feature $C_{20}H_{30}O_5$ *m/z* 349.20 RT 9.5 min assigned to putative lipoxin A5/resolving E1 (RP18 dataset).

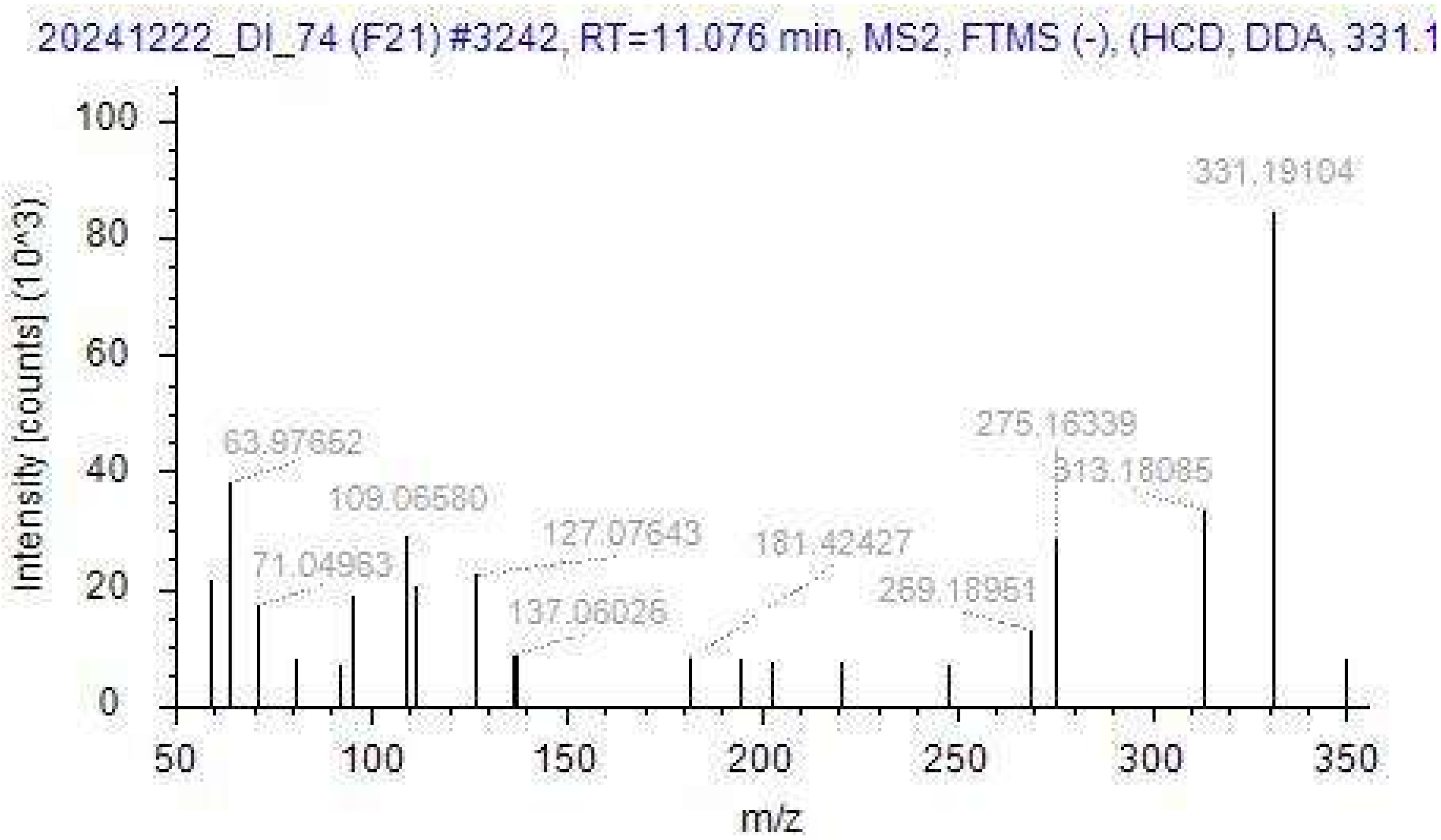

Figure S13. MS/MS spectrum for the feature $C_{20}H_{30}O_5$ *m/z* 349.20 RT 11.0 min assigned to putative delta-17-PGE1 (RP18 dataset).

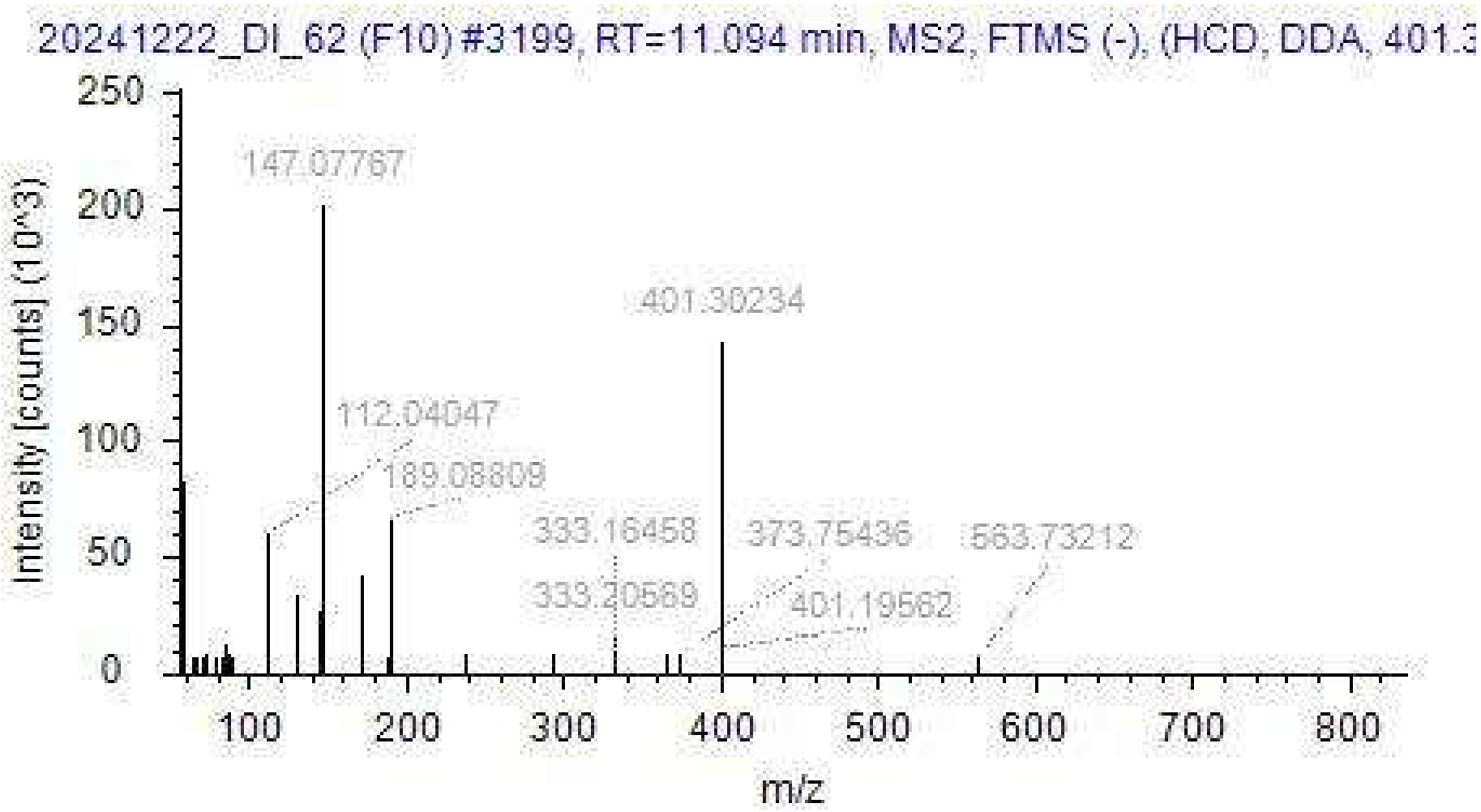

Figure S14. MS/MS spectrum for the feature $C_{21}H_{42}N_2O_5$ *m/z* 401.30 RT 11.1 min assigned to n-acyl alpha aminoacid derivative (RP18 dataset).

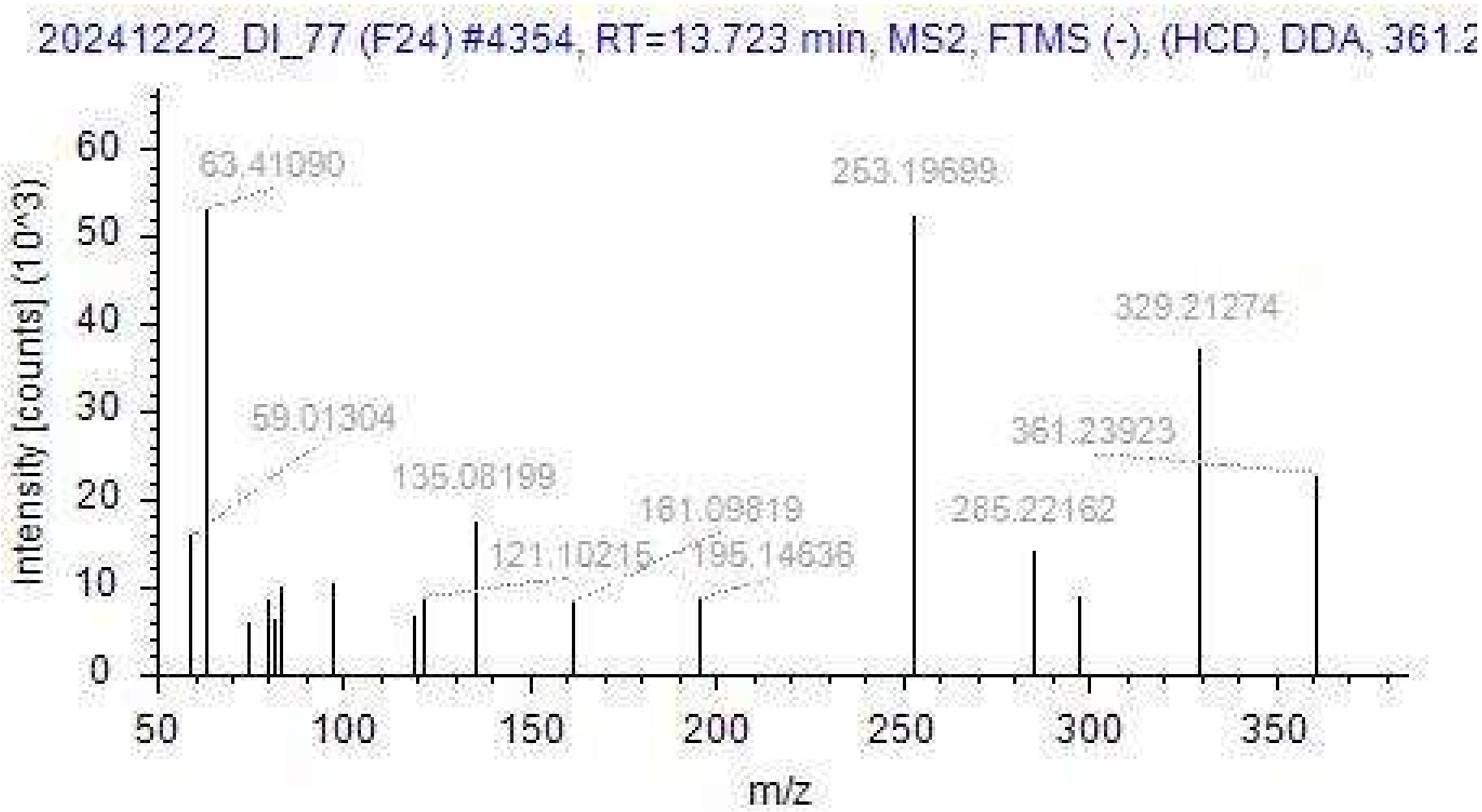

Figure S15. MS/MS spectrum for the feature $C_{22}H_{34}O_4$ *m/z* 361.23 RT 13.7 min assigned to diterpenoid derivative related to DiHDPE (RP18 dataset).

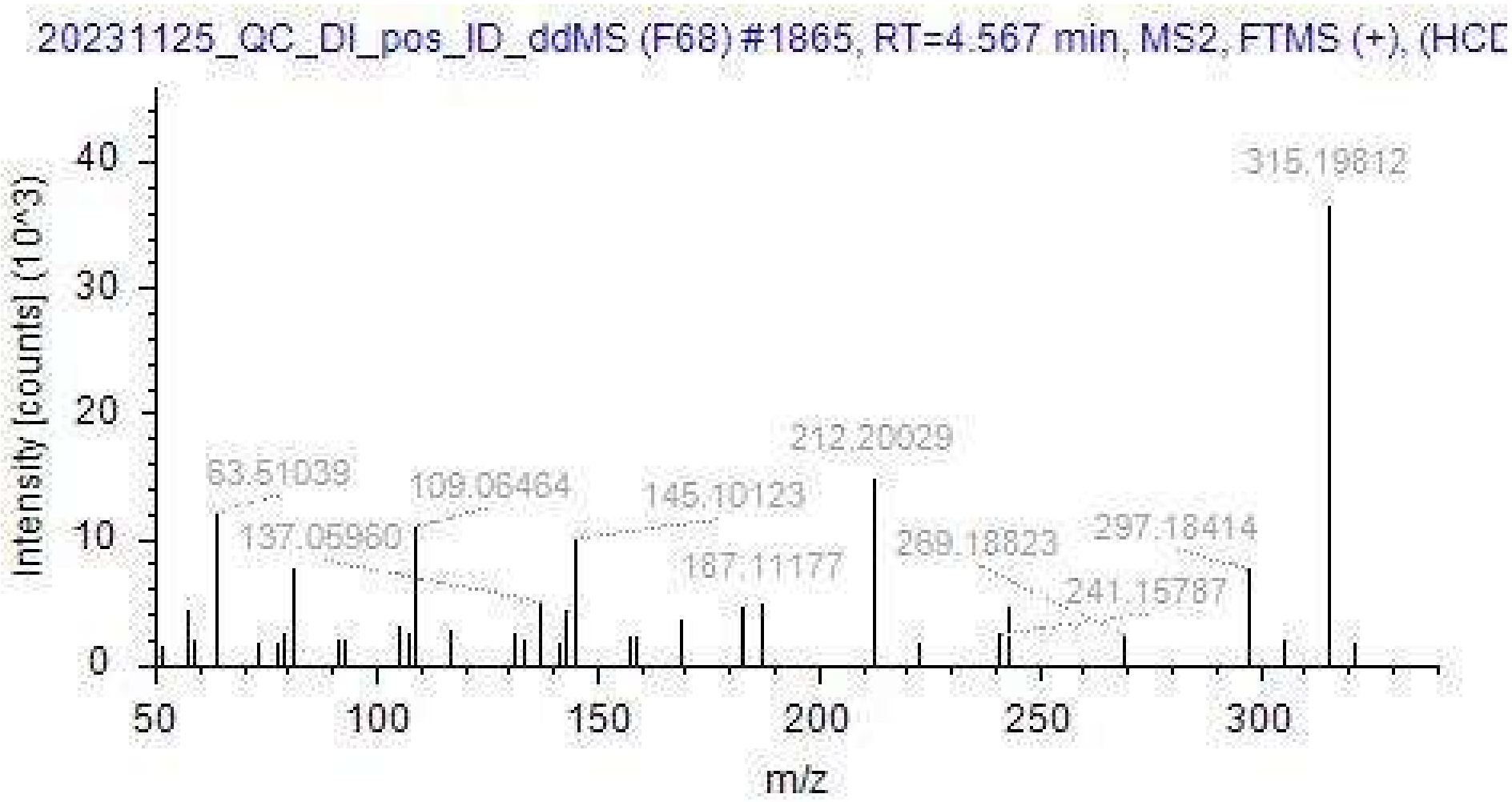

Figure S16. MS/MS spectrum for the feature $C_{20}H_{26}O_3$ *m/z* 315.19 RT 4.5 min assigned to an estrane steroid derivative (C18 dataset).

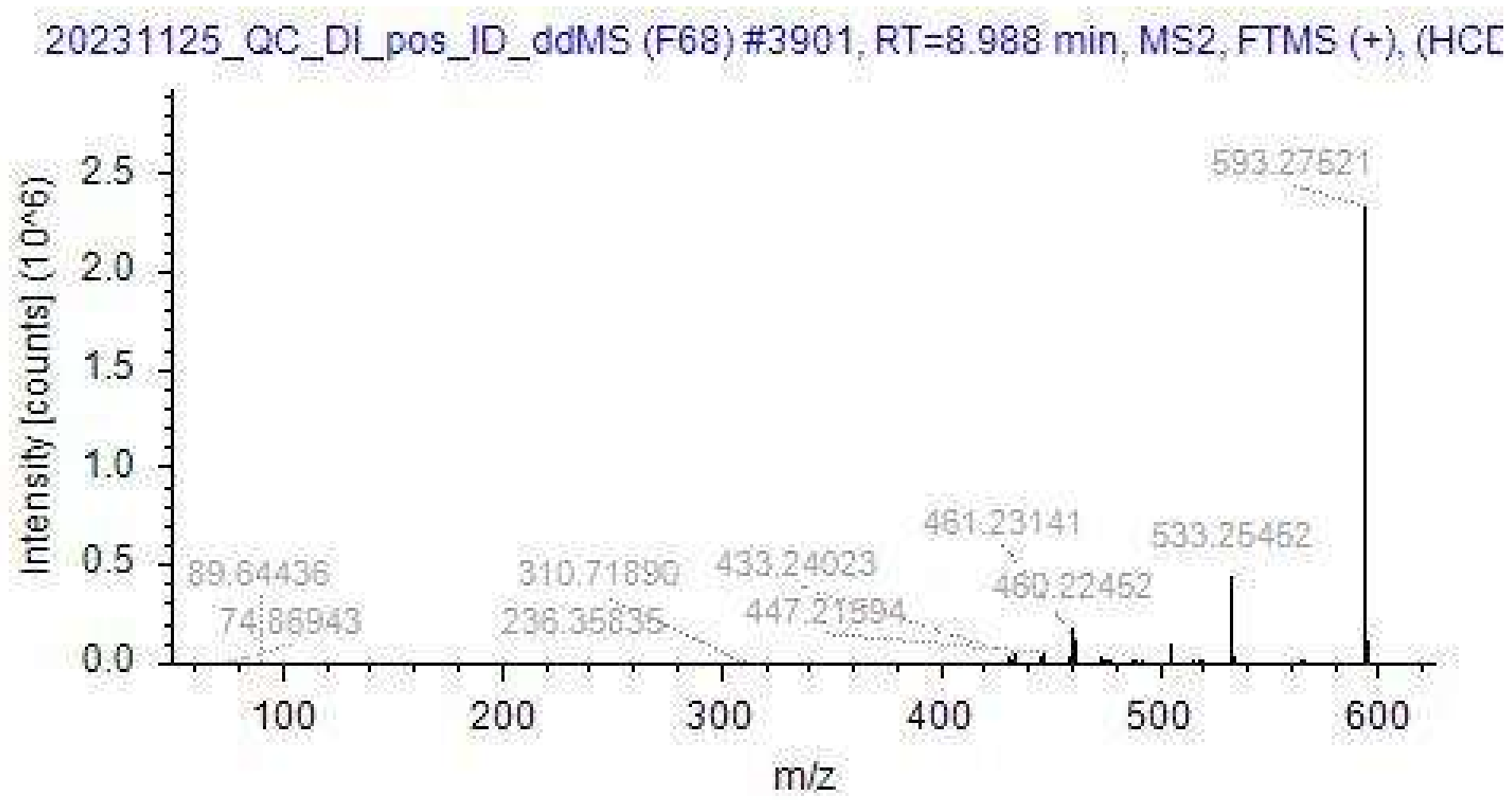

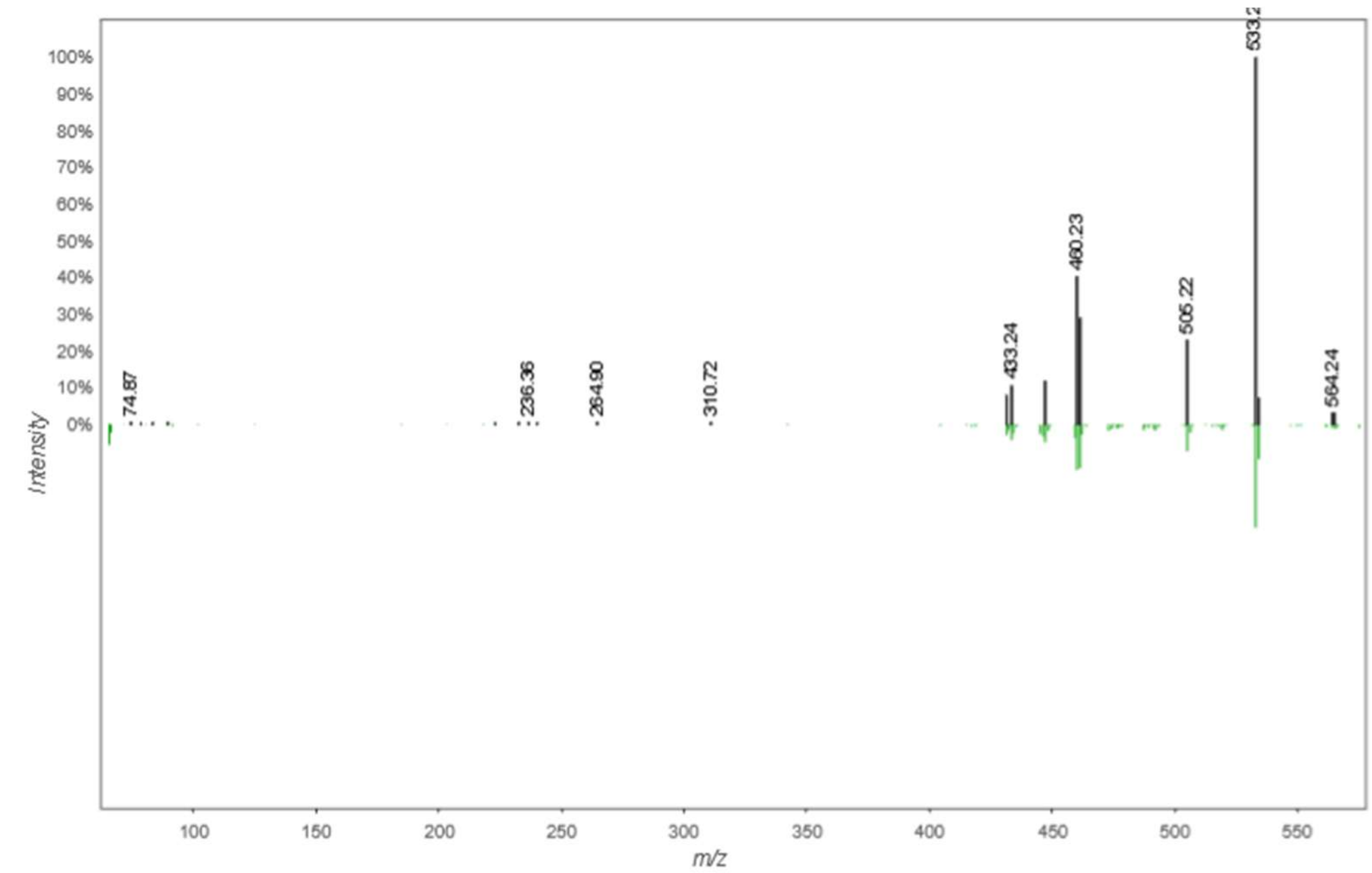

Figure S17. MS/MS spectrum for the feature $C_{35}H_{36}N_4O_5$ *m/z* 593.27 RT 8,9 min assigned to pheophorbide a (C18 dataset). Match with Phaeophorbide a with a cosine score of 0.86.

https://gnps.ucsd.edu/ProteoSAFe/gnpslibraryspectrum.jsp?SpectrumID=CCMSLIB00010128702#%7B%7D

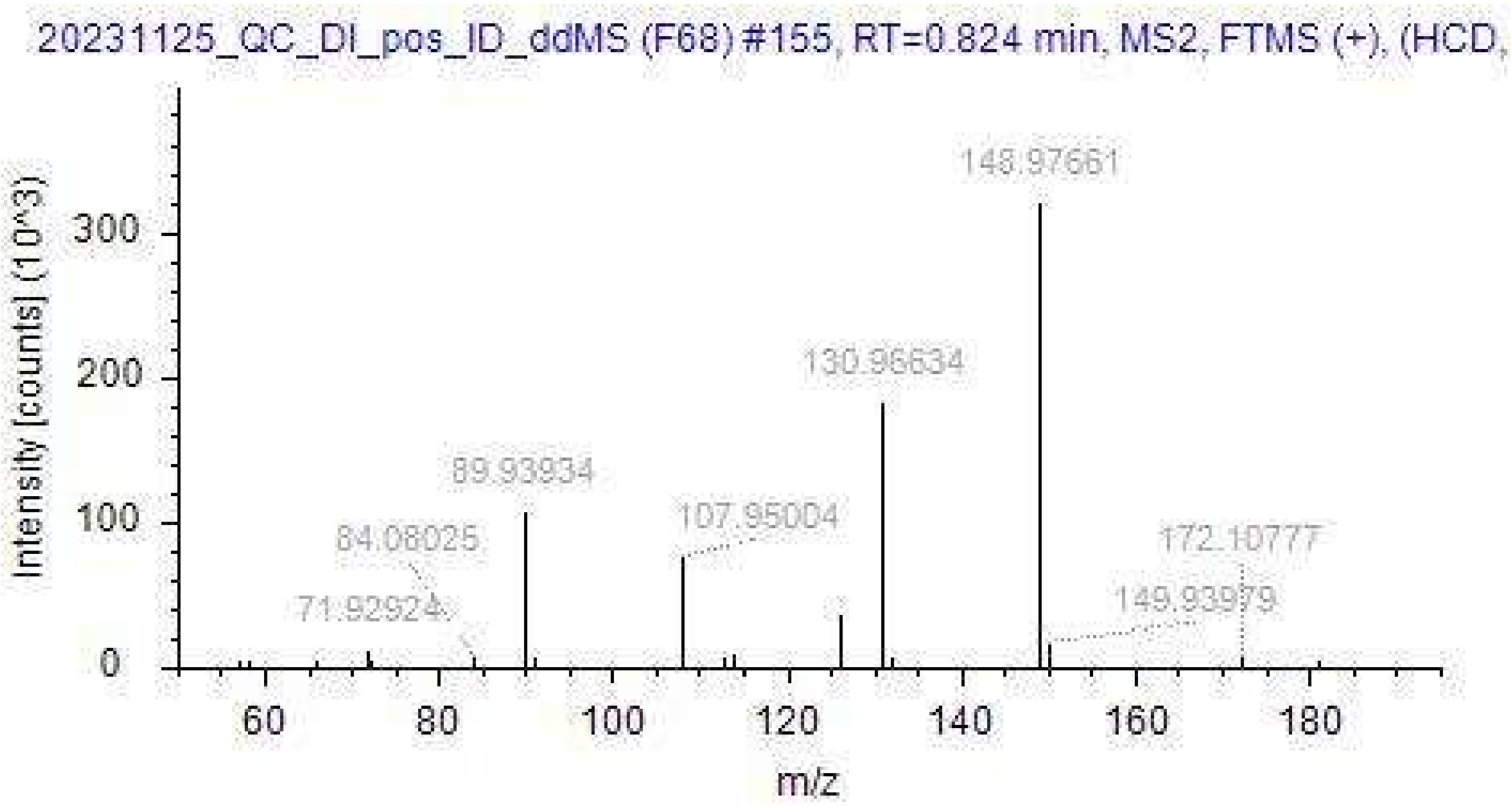

Figure S18. MS/MS spectrum for the feature $C_8H_{13}O_3$ *m/z* 172.09 RT 3.7 min assigned to butyrylhomoserine lactone derivative (C18 dataset).

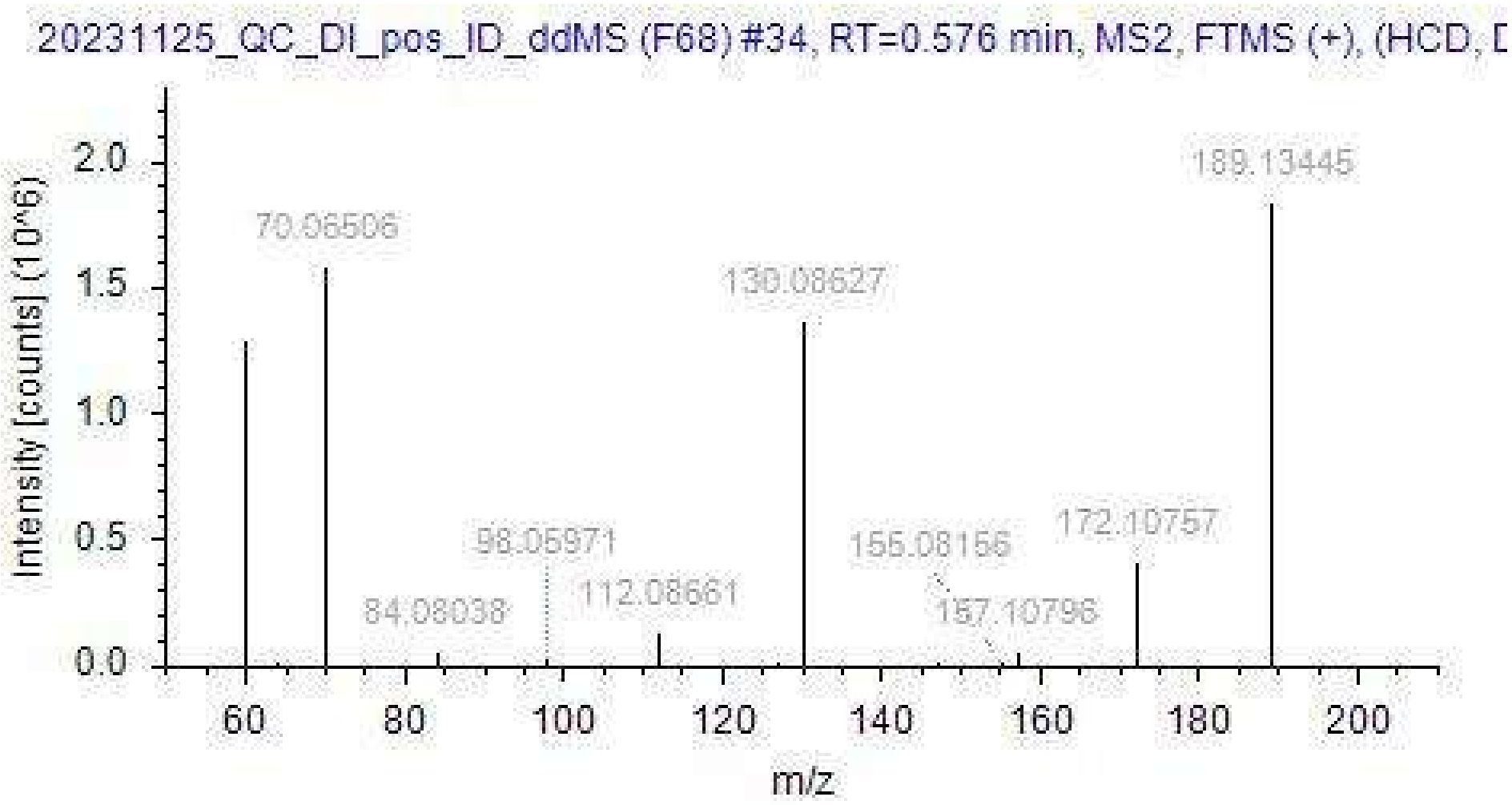

Figure S19. MS/MS spectrum for the feature $C_7H_{16}N_4O_2$ *m/z* 189.13 RT 0.5 min assigned to homoarginine (C18 dataset).

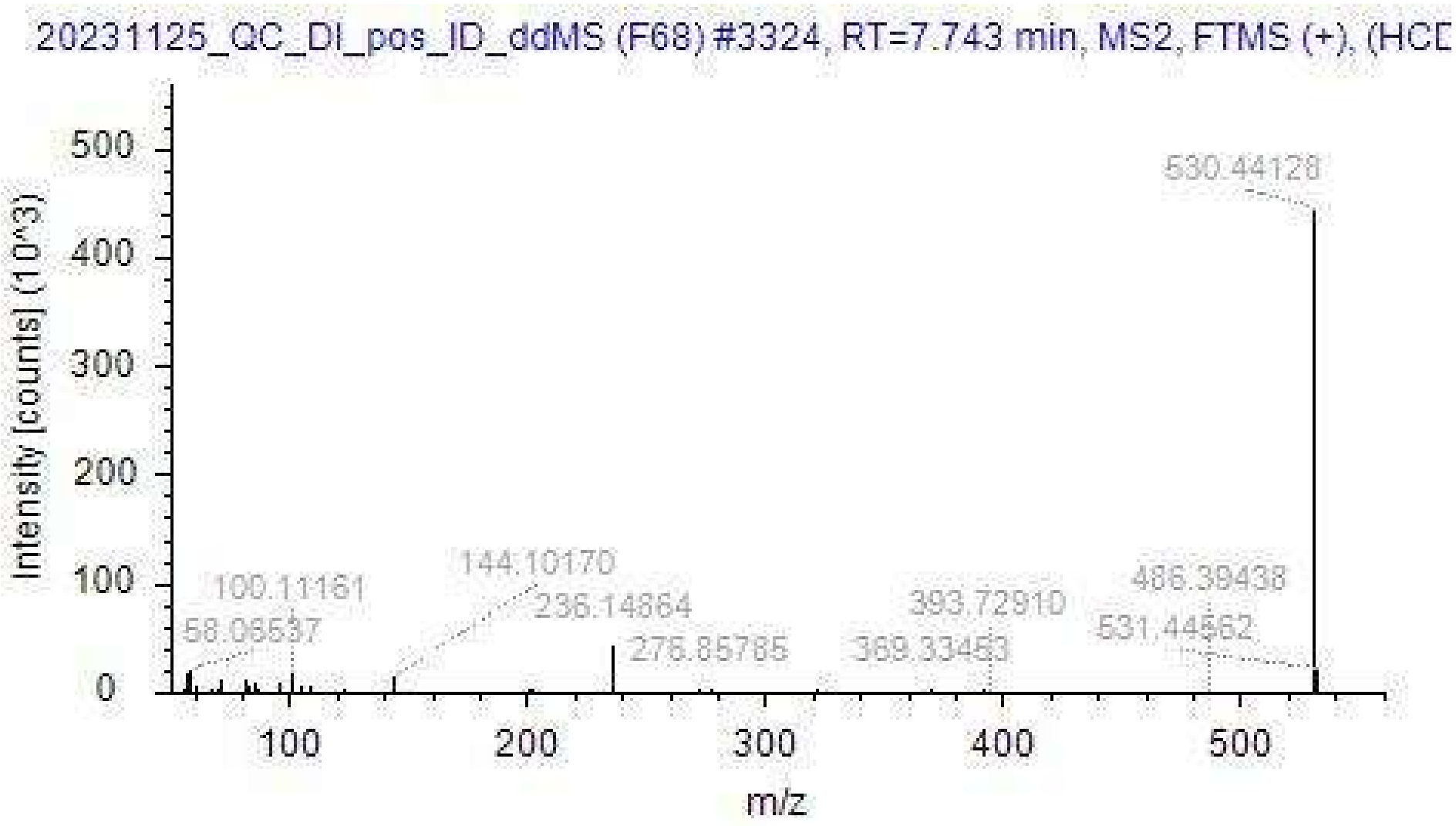

Figure S20. MS/MS spectrum for the feature $C_{30}H_{59}NO_6$ *m/z* 530.44 RT 7.7 min assigned to MGTS (20:0/0:0) (C18 dataset).

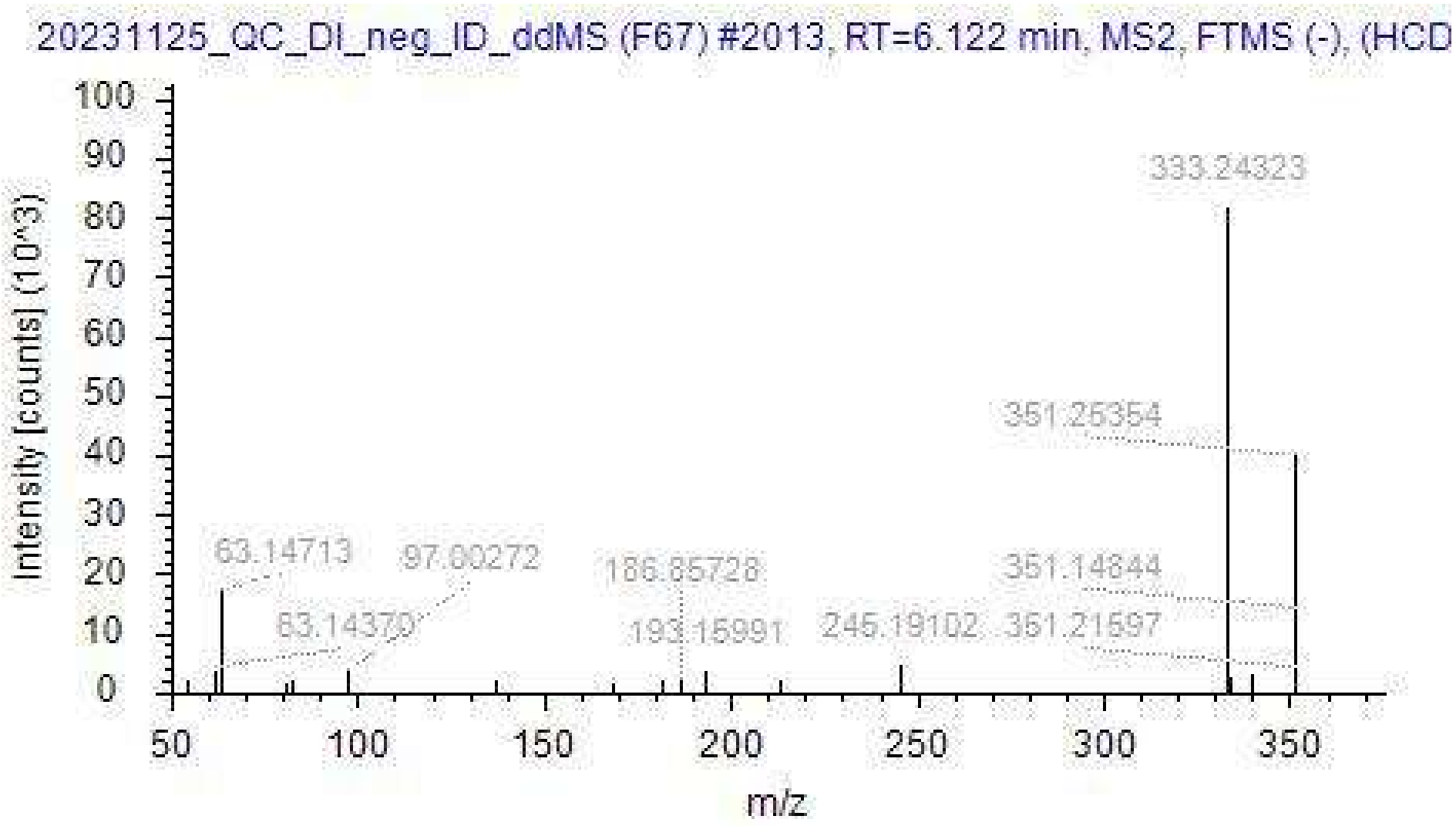

Figure S21. MS/MS spectrum for the feature $C_{21}H_{36}O_4$ *m/z* 351.25 RT 6.1 min assigned to putative ceriporic acid (C18 dataset).

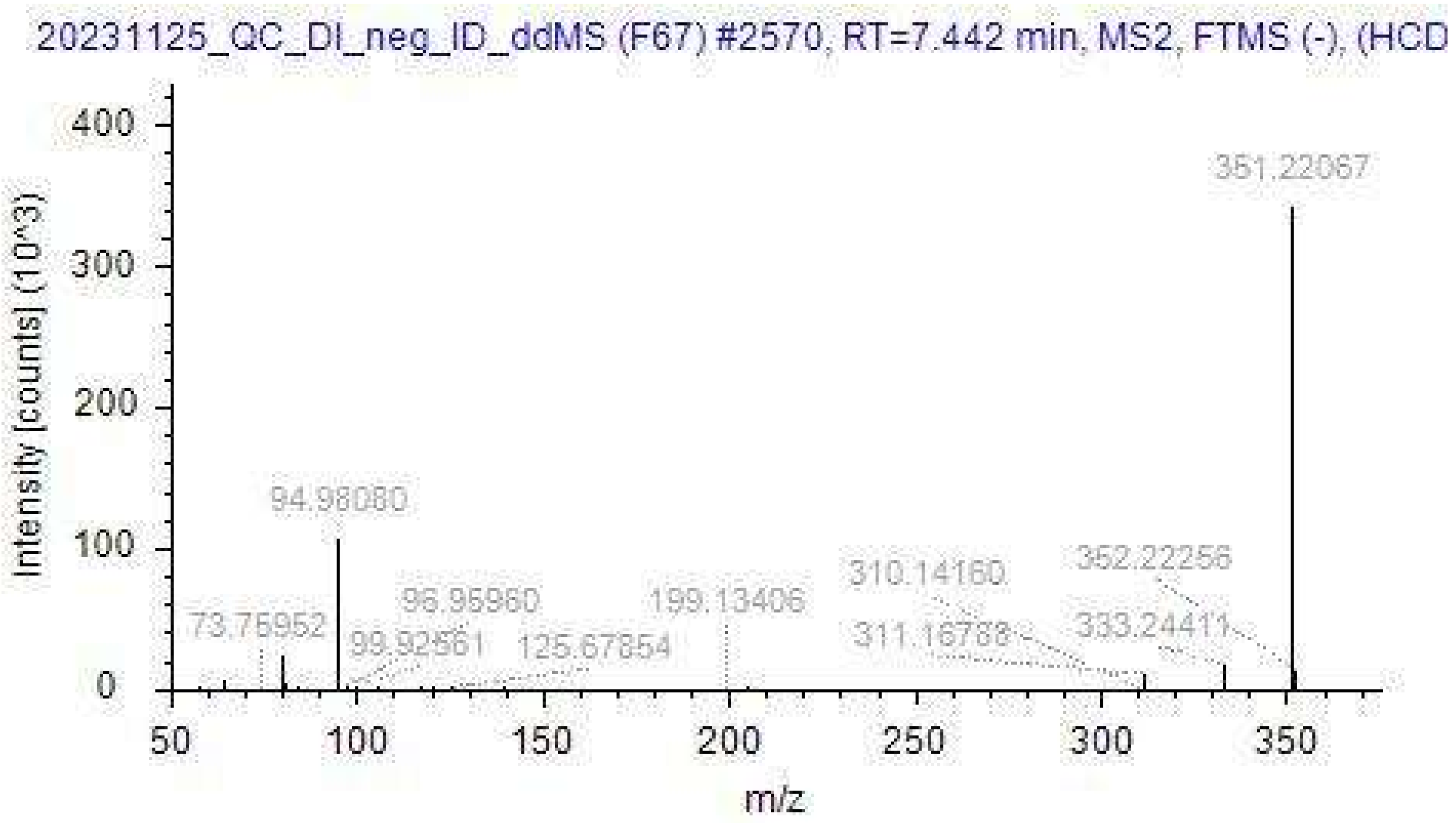

Figure S22. MS/MS spectrum for the feature $C_{21}H_{36}O_4$ *m/z* 351.25 RT 7.4 min assigned to putative chaetomellic acid (C18 dataset).

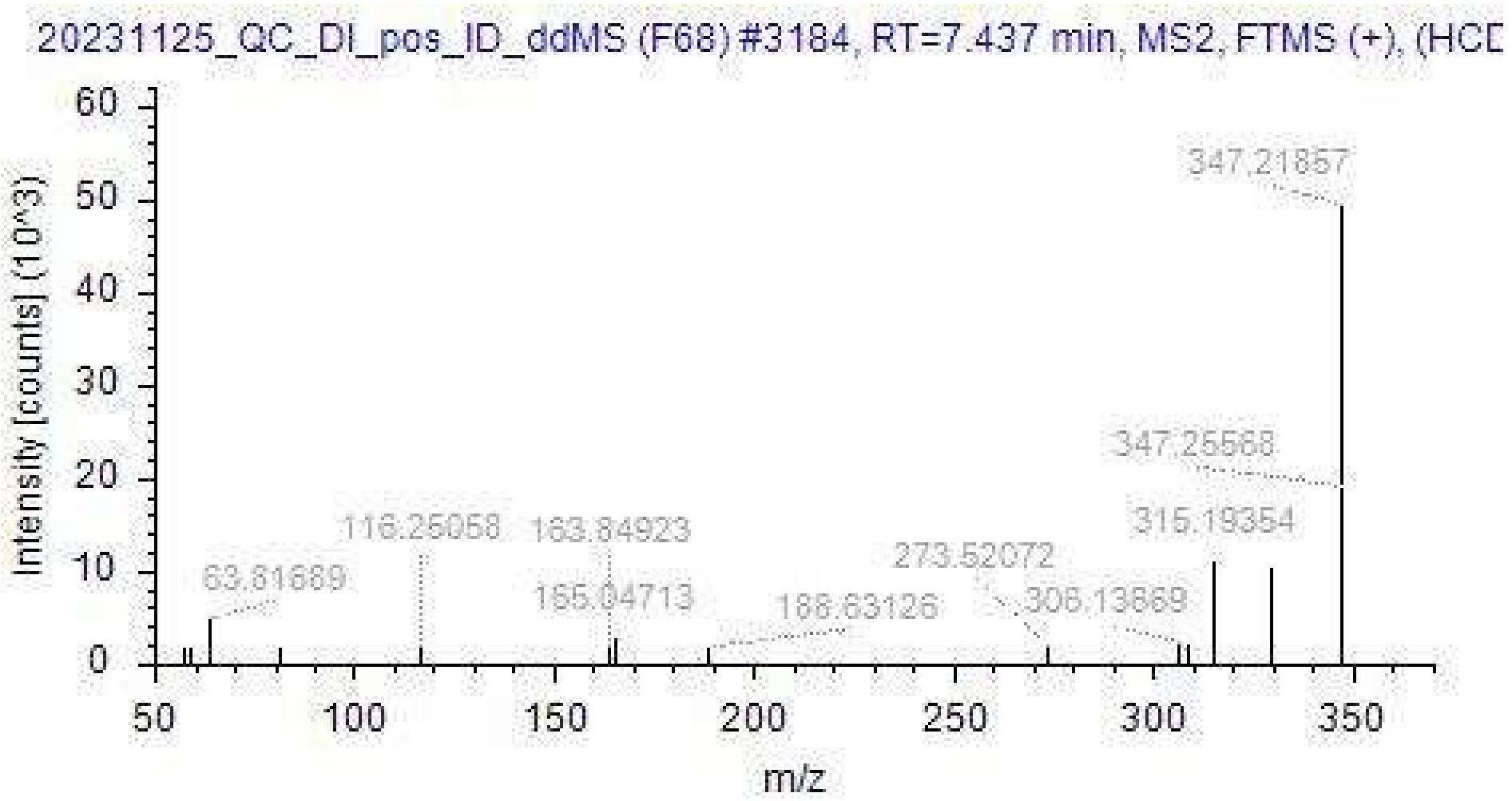

Figure S23. MS/MS spectrum for the feature $C_{21}H_{32}O_2$ *m/z* 317.24 RT 7.3 min assigned to prenol lipid derivative (C18 dataset).

Supplementary Tables

Table S1. Diatom abundances, infection prevalence and calculated log2 fold change.

Day	Temperature [°C]	Treatment	Non-infected cell abundance Mean ± SD	Infected cell abundance Mean ± SD	Unit	Infection prevalence Mean ± SD	log2 fold change in non-infected cell abundance ± SD	log2 fold change in infected cell abundance ± SD	log2 fold change in infection prevalence ± SD
1	5	Control	952±254.476		mL^{-1}		1.81±0.286		
1	10	Control	1325.75±75.780		mL^{-1}		2.23±0.132		
1	15	Control	1210±245.060		mL^{-1}		2.44±0.172		
3	5	Control	2068±110.777		mL^{-1}				
3	10	Control	2264.5±449.602		mL^{-1}				
3	15	Control	2631.5±241.068		mL^{-1}				
7	5	Control	3077.25±516.671		mL^{-1}				
7	10	Control	5341.5±272.608		mL^{-1}				
7	15	Control	5464.5±671.629		mL^{-1}				
1	5	Chytrid-infected	1413±580.506	218±50.298	mL^{-1}	15.12±6.99	1.01±0.4	1.59±0.68	2.15±0.153
1	10	Chytrid-infected	1653.75±780.595	247.25±54.22	mL^{-1}	14.03±4.38	0.73±0.403	2.46±0.481	2.217±0.44
1	15	Chytrid-infected	1230.5±622.972	387.5±114.216	mL^{-1}	26.84±11.42	1.2±0.59	2.72±0.76	2±0.64
3	5	Chytrid-infected	3151.5±800.502	439.75±112.721	mL^{-1}	12.61±3.89			
3	10	Chytrid-infected	2982±627.775	488.5±88.007	mL^{-1}	14.52±4.22			
3	15	Chytrid-infected	3627±699.221	618.5±43.193	mL^{-1}	14.9±2.99			
7	15	Chytrid-infected	3666.5±768.507	837±187.160	mL^{-1}	18.62±2.71			

Day	Temperature [°C]	Treatment	Non-infected cell abundance Mean ± SD	Infected cell abundance Mean ± SD	Unit	Infection prevalence Mean ± SD	log2 fold change in non-infected cell abundance ± SD	log2 fold change in infected cell abundance ± SD	log2 fold change in infection prevalence ± SD
7	10	Chytrid-infected	7362.25±1633.988	891.5±114.041	mL^{-1}	11.02±2.05			
7	15	Chytrid-infected	6863.5±1102.279	1224±219.340	mL^{-1}	15.25±2.88			
1	5	Oomycete-infected	1823±50.439	371.25±74.053	mL^{-1}	16.87±3.09	1.33±0.165	1.05±0.096	1.34±0.4
1	10	Oomycete-infected	1790.75±292.635	472.25±34.250	mL^{-1}	21.31±4.52	0.65±0.231	2.01±0.309	1.8±0.535
1	15	Oomycete-infected	3412±205.468	459.25±62.344	mL^{-1}	11.86±1.42	1.89±0.458	1.36±0.16	2.92±0.203
3	5	Oomycete-infected	3477.25±605.683	462.5±118.647	mL^{-1}	11.66±1.98			
3	10	Oomycete-infected	3246±316.243	915±126.879	mL^{-1}	22.19±4.3			
3	15	Oomycete-infected	4160.75±422.585	1214.25±49.161	mL^{-1}	22.7±2.15			
7	5	Oomycete-infected	2731.75±675.408	869.25±172.976	mL^{-1}	24.13±0.9			
7	10	Oomycete-infected	6599.5±785.727	1548±321.138	mL^{-1}	19.02±3.55			
7	15	Oomycete-infected	5152±1375.918	2995.25±217.855	mL^{-1}	37.69±7.75			

Table S2. Results of one-way ANOVA and Tukey HSD post hoc tests assessing the effect of temperature on log_2 fold change in *Ulnaria* abundances and infection prevalence. ANOVA results include the degrees of freedom (df), mean square (MS), F-statistic (F), and p-value (p). Tukey HSD post hoc comparisons indicate significant pairwise differences between temperature treatments. Significant results ($p < 0.05$) are highlighted in bold.

Uln treatment								
Dependent variable	df	MS	F	p	sign	Tukey HSD	95% C.I.	p adj
Non-infected *Ulnaria*	2	2.47	62.99	**<0.001**	***	10-5	[0.28, 0.55]	**<0.001**
						15-5	[0.49, 0.77]	**<0.001**
						15-10	[0.08, 0.35]	**0.001**
**Chy treatment**								
Dependent variable	df	MS	F	p	sign	Tukey HSD	95% C.I.	p adj
Non-infected *Ulnaria*	2	4.82	14.49	**<0.001**	***	10-5	[0.31, 1.32]	**<0.001**
						15-5	[0.57, 1.57]	**<0.001**
						15-10	[-0.24, 0.75]	0.43
Infected *Ulnaria*	2	0.19	1.12	0.33		10-5	[-0.28, 0.44]	0.86
						15-5	[-0.5, 0.22]	0.62
						15-10	[-0.57, 0.14]	0.31
Infection prevalence	2	0.9	5.04	0.01	*	10-5	[-0.62, 0.11]	0.23
						15-5	[-0.15, 0.59]	0.32
						15-10	[0.11, 0.84]	**0.008**
**Oom treatment**								
Dependent variable	df	MS	F	p	sign	Tukey HSD	95% C.I.	p adj
Non-infected *Ulnaria*	2	3.81	118.3	**<0.001**	***	10-5	[0.82, 1.14]	**<0.001**
						15-5	[0.15, 0.46]	**<0.001**
						15-10	[-0.83, -0.52]	**<0.001**
Infected *Ulnaria*	2	10.78	83.68	**<0.001**	***	10-5	[0.17, 0.8]	**0.002**
						15-5	[1.31, 1.93]	**<0.001**
						15-10	[0.82, 1.45]	**<0.001**
Infection prevalence	2	5.88	73.5	**<0.001**	***	10-5	[-0.91, -0.41]	**<0.001**
						15-5	[0.32, 0.82]	**<0.001**
						15-10	[0.99, 1.48]	**<0.001**

Table S3. Samples metadata list for metabolomics analysis. NA: no data

Sample code	Temperature (°C)	Sample label	Parasite treatment	Extract type	Cell density (cell per mL)	Normalization factor
40	5	blank medium	blank	endometabolome	NA	NA
41	5	Uln-H1	Control	endometabolome	3010	0.24
42	5	Uln-H2	Control	endometabolome	2773	0.22
43	5	Uln-H3	Control	endometabolome	3728	0.29
44	5	Uln-H4	Control	endometabolome	3143	0.25
45	5	Uln-INF1	Chytrid-infected	endometabolome	4115	0.32
46	5	Uln-INF2	Chytrid-infected	endometabolome	4988	0.39

47	5	Uln-INF3	Chytrid-infected	endometabolome	5213	0.41
48	5	Uln-INF4	Chytrid-infected	endometabolome	4225	0.33
49	5	Uln-O1	Oomycete-infected	endometabolome	4441	0.35
50	5	Uln-O2	Oomycete-infected	endometabolome	2852	0.22
51	5	Uln-O3	Oomycete-infected	endometabolome	3334	0.26
52	5	Uln-O4	Oomycete-infected	endometabolome	3777	0.30
53	10	blank medium	blank	endometabolome	NA	NA
54	10	Uln-H1	Control	endometabolome	5367	0.42
55	10	Uln-H2	Control	endometabolome	5273	0.41
56	10	Uln-H3	Control	endometabolome	6337	0.50
57	10	Uln-H4	Control	endometabolome	5570	0.44
58	10	Uln-INF1	Chytrid-infected	endometabolome	11521	0.90
59	10	Uln-INF2	Chytrid-infected	endometabolome	8694	0.68
60	10	Uln-INF3	Chytrid-infected	endometabolome	12469	0.98
61	10	Uln-INF4	Chytrid-infected	endometabolome	7746	0.61
62	10	Uln-O1	Oomycete-infected	endometabolome	7423	0.58
63	10	Uln-O2	Oomycete-infected	endometabolome	9188	0.72
64	10	Uln-O3	Oomycete-infected	endometabolome	8302	0.65
65	10	Uln-O4	Oomycete-infected	endometabolome	7677	0.60
66	15	blank medium	blank	endometabolome	NA	0.00
67	15	Uln-H1	Control	endometabolome	5307	0.42
68	15	Uln-H2	Control	endometabolome	6564	0.51
69	15	Uln-H3	Control	endometabolome	6903	0.54
70	15	Uln-H4	Control	endometabolome	5423	0.42
71	15	Uln-INF1	Chytrid-infected	endometabolome	9271	0.73
72	15	Uln-INF2	Chytrid-infected	endometabolome	9633	0.75
73	15	Uln-INF3	Chytrid-infected	endometabolome	12763	1.00
74	15	Uln-INF4	Chytrid-infected	endometabolome	8792	0.69
75	15	Uln-O1	Oomycete-infected	endometabolome	9995	0.54
76	15	Uln-O2	Oomycete-infected	endometabolome	6980	0.30
77	15	Uln-O3	Oomycete-infected	endometabolome	7488	0.17
78	15	Uln-O4	Oomycete-infected	endometabolome	8446	0.11
